# The Molecular Pathology of Blood Cancer: A Comprehensive Review of Chromosome and Genetic Abnormalities and Their Clinical Utility

**DOI:** 10.3389/bjbs.2025.14745

**Published:** 2026-04-20

**Authors:** N. McCaul, R. J. Bingham, A. D. Blann

**Affiliations:** Department of Applied Sciences, Huddersfield University, Huddersfield, United Kingdom

**Keywords:** blood cancer, leukaemia, lymphoma, molecular pathology, myeloma

## Abstract

Molecular pathology has, without a doubt, transformed the field of blood cancer. Thanks to pioneers such as Sanger and Mullis, techniques such as next- and third-generation sequencing, and whole exome sequencing have, alongside a revolution in bioinformatics, determined abnormalities in chromosomes and genes with exquisite sensitivity and specificity. These have contributed considerably not just to our understanding of the cell biology, aetiology, classification, and pathophysiology of blood cancer, but also to its diagnosis and management. Good examples of this include the ability to recognise and treat cases of aberrant tyrosine kinase activity with targeted inhibitors and the recognition that certain abnormalities are linked to a more severe outcome, so that focused treatment can begin. This review catalogues these discoveries and describes how they contribute to our understanding of, and thus the treatment of, lymphoma, leukaemia, myeloma, and other myeloproliferative, erythroid, megakaryocytic, and lymphoid neoplasms. Inevitably, as new techniques are developed, we can expect further advances in biomedical science in all aspects of blood cancer.

## Introduction

Each year, the UK’s Office for National Statistics (ONS) publishes data on the leading causes of death [1]. In 2023, of the 580,108 deaths in England and Wales, 152,418 (26.3%, the leading cause) were due to malignant neoplasms, and of these, those of the lung, bronchus and trachea were the most common (27,856: 18.32%), followed by cancer of the colon and rectum (15,527: 10.2%). The third most frequent cause of a cancer death is cancer of the lymphoid, haematopoietic and related tissues, i.e., blood cancer, with 12,193 deaths, exceeding those of prostate (11,072) and breast cancers (9,973). As genetics are implicated in the aetiology of cancer [2], this narrative review will summarise leading aspects of the role of chromosomal and genetic molecular pathology in the diagnosis and treatment of blood cancer. We will address this issue first with a historical perspective and then move on to the role of genetics in blood cancer subtypes. In doing so, we will adopt the standard shorthand notations for describing the leading chromosomal and genetic abnormalities (Table 1).

**TABLE 1:** Shorthand descriptors of the most common chromosomal and genetic abnormalities.

Shorthand example	Meaning
+8	An extra copy of chromosome 8, and so a trisomy
−7	Loss of an entire chromosome 7, and so a monosomy
−13q	Loss of the q arm of chromosome 13
i(17q)	Loss of the p arm of chromosome 17 and its replacement by the q arm, and so two q arms.
t(9;22)(q34;q11)	Translocation of q34 from chromosome 9 to the q11 region of chromosome 22
del(17p)	Deletion of the p arm of chromosome 17
inv(3)(q21; q26)	Inversion of section q21 to q26 of chromosome 3
iAMP21	Intrachromosomal amplification of chromosome 21
*BCR::ABL1*	Fusion gene of *BCR* and *ABL1*

## Historical Perspective

Given the ease with which a sample can be obtained, blood has long been the tissue of choice for numerous studies in genetics. Miescher is credited with the first description, in 1871, of a substance he named nuclein, obtained from the nuclei of white blood cells [3, 4]. Cancer of the blood (as leucocythaemia) was specifically described by Bard in 1888, and bone marrow cancer was described by Stolte in 1948 [5, 6]. In 1954, Lange et al. concluded that ionising radiation is a leading external cause of leukaemia [7], while a further key report on leukaemogenesis used cytogenetics to demonstrate chromosomal abnormalities in chronic granulocytic leukaemia [8]. Baikie et al. subsequently reported chromosomal abnormalities in a variety of acute leukaemias [9]. These studies were among the first to lay the foundations for understanding the role of DNA in cancer.

Analytical developments during the 1970s, such as DNA/RNA hybridisation, allowed for the detection of RNA from leukaemias and lymphomas that exhibited sequence homology to murine leukaemia virus [10]. This allowed Gallo and Wong-Stall to hypothesise the involvement of viral oncogenes (first described by Huebner and Todaro in 1969) in leukaemogenesis [11, 12]. Rowley reported abnormalities in a number of chromosomes in various blood cancers, including additional copies of chromosome 1, i(17q) and the translocation t(15;17)(q22;q21), which we now know brings together the *PML* and *RARA* genes and will be discussed below [13]. During the 1980s, an increasing number of genetic analyses reported translocations with carcinogenic potential, such as the transfer of an oncogene next to an immunoglobulin heavy chain gene, changes that were subsequently found in numerous lymphomas and leukaemias. The leading example of this is from 1982, with the translocation of a form of *MYC* (i.e., *c-MYC*, subsequently shown to be a leading oncogene) next to a gene for part of an immunoglobulin molecule linked to Burkitt lymphoma [14]. In 1987, Weiss et al. reported the presence of the Epstein-Barr virus (EBV) in Hodgkin’s disease tissues. This was expanded upon in 1990 by Herbst and colleagues, who extracted DNA from paraffin-embedded tissues and used *in situ* hybridisation with a probe of sections of the EBV genome. Both studies pointed to a potential causal effect and demonstrated the feasibility of probing archived tissues [15].

One of the most significant examples of the power of molecular pathology is the demonstration that the genetic lesion in the Philadelphia chromosome [8, 9] is a translocation of part of one chromosome to another [16]. The decade that followed saw the now classic report that this transfer brings together two otherwise non-pathological genes (*BCR* and *ABL1*) to form a new oncogene (i.e., a neo-oncogene) that causes chronic myeloid leukaemia, an important point that will be discussed below. By the mid-1980s, radiolabelled probes and hybridising Southern blotting were being used to demonstrate the amplification of oncogenes such as *c-myc* and *KRAS* (described by some as *c-MYC* and *k-Ras,* respectively) in lung and other cancers [17].

**FIGURE 1:**
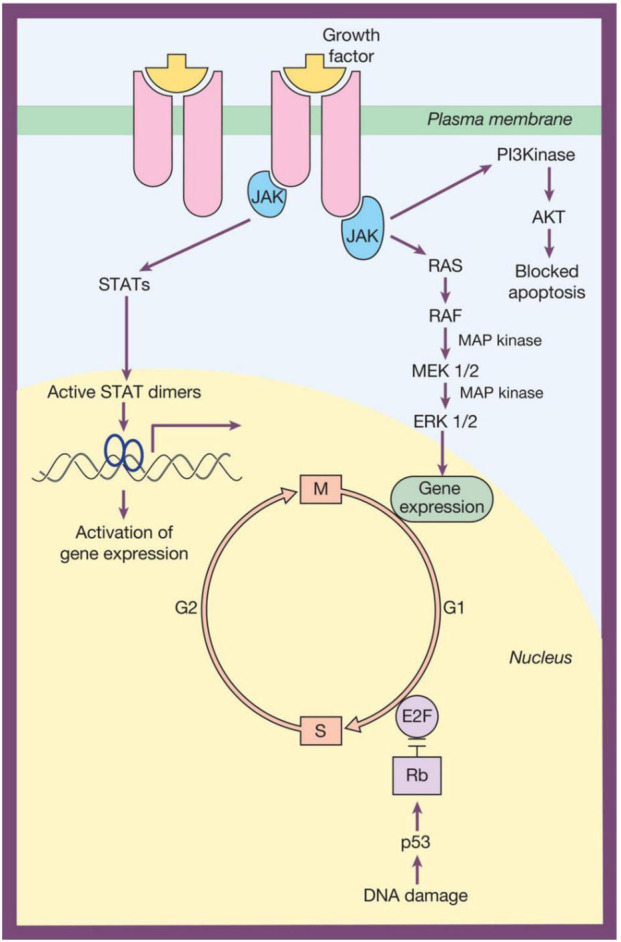
Signal transduction pathways. In this simple representation, at the top, the docking of a ligand such as a growth factor (others include certain interleukins and interferons) with its cell-surface receptor leads to the activation of kinases, such as those of the JAK family. These enzymes then activate second messengers downstream, including those of the STAT and RAS/RAF/MEK/ERK pathways, thereby becoming involved in the regulation of the cell cycle. A second pathway, bottom right, involves the activation of *TP53* following DNA damage, where its protein product, p53, interacts with transcription factors that also have an influence on the progression of the cell cycle. Reproduced with permission from Figure 1.8 "Control of hemopoiesis by growth factors" by A. Victor Hoffbrand, Pratima Chowdary, Graham P. Collins and Justin Loke.

In 1990, Becher et al. reported a series of cases of patients with a Philadelphia-negative leukaemia, where the sole chromosome structural abnormality was an i17q, although there was also +8, +17 or +19 in some subjects [18]. By the middle of the decade, all forms of blood cancer had been linked to a genetic lesion, while technological advances in molecular genetics were being used in routine clinical practice [19]. An example of this is the use of PCR technology to probe for minimal/measurable residual disease (MRD) in the treatment of acute lymphoblastic leukaemia [20]. The present millennium has brought, as with so many other pathologies, a revolution in molecular pathology, initially with the development of next-generation sequencing (NGS) and subsequently with third-generation sequencing, an example being the discovery of other aspects of molecular genetics, such as non-coding RNA. These and other advances have led directly to our understanding of the complex nature of signal transduction and so how abnormalities can lead to malignancies [21, 22] (Figure 1). Recent reviews of the impact of molecular pathology on blood cancer include those by Behrens and colleagues and Kwon and Yeung [23, 24].

## The Epidemiology of Blood Cancer

The World Health Organization publishes data on the incidence and mortality of the leading forms of blood cancer both globally and by nation [25] (Table 2). The global and UK data sets are broadly in agreement, showing that the incidence frequencies are both lymphoma > leukaemia > myeloma, while the outcome indices (mortality/incidence, where higher = least favourable) are myeloma > leukaemia > lymphoma. However, the outcome indices are all worse globally. There are no incidence or mortality data on the remaining rare causes of blood cancer deaths, such as those caused by myeloproliferative neoplasms. Nevertheless, we will adopt the WHO classification system for the remainder of this review.

**TABLE 2:** Incidence and mortality of blood cancers.

​	Global			UK		
	Incidence	Mortality	Outcome index	Incidence	Mortality	Outcome index
All blood cancers	1,311,104	700,205	0.534	35,576	14,934	0.420
Leukaemia	487,294	305,405	0.627	10,755	5,335	0.496
Lymphoma	635,858	273,412	0.430	18,317	5,981	0.326
Myeloma	187,952	121,388	0.646	6,504	3,618	0.556

Data are the raw numbers of patients, and the outcome index is the mortality/incidence rate. Source: WHO.

## Lymphoma

Quite reasonably, lymphoma can be regarded as a solid-organ neoplasm, with diagnosis relying primarily on conventional histology and immunocytochemistry with cell-specific CD markers on material obtained from a biopsy or a fine-needle aspirate. With its primary site in the lymph nodes, it could be argued that lymphoma is not a blood cancer, although malignant lymphoma cells may appear in the blood in advanced stages of the disease. Nevertheless, many consider the leading document regarding the molecular pathology of lymphoma to be the 2022 5^th^ Edition of the WHO classification of haematolymphoid tumours, focusing on lymphoid neoplasms [26], although there are others [27, 28].

The literature on and clinical management of this cancer are dominated by three groupings: precursor (immature) B-cell malignancies and two groups of mature B-cell cancers–long-established Hodgkin lymphoma (HL) [29, 30], and non-Hodgkin lymphoma (NHL), which are referred to separately by the WHO and other bodies (Tables 3, 4). Hodgkin lymphoma is defined by the presence of Reed-Sternberg cells, which are generally defined morphologically by standard histology (e.g., haematoxylin and eosin) as large, malignant B lymphocytes with atypical bi-lobed nuclei. A further tool is to use immunocytochemistry with markers such as CD15 and CD30. HL was linked to 290 deaths in England and Wales in 2023 (6% of lymphoma deaths), while NHL, defined by the absence of Reed-Sternberg cells, was linked to 4,555 deaths (94% of lymphoma deaths) [1]. Data from the WHO reports the global age-standardised incidence rate in the UK for Hodgkin lymphoma is 2.8/100,000, with a mortality rate of 0.21/100,000. In contrast, the respective rates for NHL are 11.2/100,000 and 2.8/100,000, respectively [25]. The majority of lymphomas originate from malignant B lymphocytes, so a focus on immunoglobulin genes is important, and this is where we shall begin, moving on later to those of malignant T and NK cells.

**TABLE 3:** WHO classification of B-cell lymphoid neoplasms.

Precursor B-cell neoplasms
• B-cell lymphoblastic leukaemias/lymphomas
Mature B-cell neoplasms
• Hodgkin lymphoma
• Burkitt lymphoma
• Large B-cell lymphomas
• Pre-neoplastic and neoplastic small lymphocytic proliferations
• Splenic B-cell lymphomas and leukaemias
• Lymphoplasmacytic lymphoma
• Marginal zone lymphoma
• Follicular lymphoma
• Cutaneous follicle centre lymphoma
• Mantle cell lymphoma
• Transformations of indolent B-cell lymphomas
• KSHV/HHV8-associated B-cell lymphoid proliferations and lymphomas
• Lymphoid proliferations and lymphomas associated with immune deficiency and dysregulation
Plasma cell neoplasms and other diseases with paraproteins
• Plasma cell neoplasms and other diseases with paraproteins
• Diseases with monoclonal immunoglobulin deposition
• Heavy chain diseases
• Plasma cell neoplasms

**TABLE 4:** WHO classification of T and NK-cell lymphoid neoplasms.

Tumour-like lesions with T-cell predominance
Precursor T-cell neoplasms
Mature T-cell and NK-cell neoplasms
• Mature T-cell and NK-cell leukaemias
• Primary cutaneous T-cell lymphomas
• Intestinal T-cell and NK-cell lymphoid proliferations and lymphomas
• Hepatosplenic T-cell lymphoma
• Anaplastic large cell lymphoma
Nodal T-follicular helper (TFH) cell lymphoma
• Nodular TFH cell lymphoma
• Other peripheral T-cell lymphomas
• EBV-positive NK/T-cell lymphomas
• EBV-positive T- and NK-cell lymphoid proliferations and lymphomas of childhood

### Precursor B-Cell Neoplasms

This short section is dominated scientifically by the recognition of the close aetiological relationship between certain lymphomas and leukaemias, in that the same chromosomal and genetic aberrations can often be detected in these lymphoblastic malignancies. This has prompted a specific section in the WHO guidelines [25], which focuses on hypo- and hyper-diploidy, such as +21, fusions between genes such as *BCR* and *ABL1*, and rearrangements in genes such as *KMT2A* (Table 5) [31–33]. Several of these genes will re-emerge in subsequent sections, such as B-acute lymphoblastic leukaemia with t(1;19)(q23;p13.3) forming *TCF3::PBX1*. This is significant for leukaemic transformation due to its links to *ROR1,* which codes for a receptor tyrosine kinase whose excess activity may promote malignant cell survival [34, 35]. Table 5 summarises the main genetic abnormalities in precursor B-cell neoplasms.

**TABLE 5:** Genes of interest in precursor B-cell neoplasms.

Gene	Location	Product
*BCR*	22q11.23	Serine/threonine-protein kinase and a guanine nucleotide exchange factor
*ABL1*	9q34.12	A tyrosine kinase
*ETV6*	12p13.2	Erythroblast transformation specific protein: A transcription factor
*RUNX1*	21q22.12	Runt-related transcription factor 1
*TCF3*	19p13.3	A transcription factor
*PBX1*	1q23.3	Pre-B-cell leukaemia transcription factor 1
*HLF*	17q22	Hepatic leukaemia factor – a transcription factor
*IGH*	14q32.33	Immunoglobulin heavy chain locus
*IL3*	5q31.1	Interleukin 3
*KMT2A*	11q23.3	Lysine methyltransferase 2A

### Hodgkin Lymphoma (HL)

While Smithers summarised the genetic/familial aspects of HL in 1967, antibodies to the Epstein-Barr virus (EBV) were recognised in the 1970s, and the potential of EBV as a true HL oncovirus emerged in the 1980s [36–38]. However, the direct effect of the virus on the aetiology of the lymphoma is incomplete, as approximately 95% of the healthy UK population is infected with no apparent ill effects [39]. This decade also saw the development of methods such as cytogenetics, DNA hybridisation, and flow cytometry to report aneuploidy, rearrangements in immunoglobulin and T-cell receptor genes, and abnormalities at sites such as 11q23, which is now known to be the location of proto-oncogene *ETS1*. Others reported the involvement of 14q32, the site of the immunoglobulin heavy chain (*IgH*) locus, with 8q22-24, the location of the *MYC* locus, and with 18q21.33, the location of *BCL2*, which codes for an apoptosis regulator. Translocations bringing *IGH* adjacent to these two oncogenes in t(11;14)(q23;q32) and t(11;18)(q32;q21) provide the aetiological rationale for the malignant transformation of the B lymphocyte that forms the basis of the lymphoma [40–43]. Schouten and colleagues used cytogenetics to probe tissues from 29 HL patients, reporting aneuploidy in 13 of them, most commonly involving chromosomes 5, 9, 15, 18, 22 and X [44].

In the present millennium, Enciso-Mora and colleagues used the power of an NGS genome-wide association study (GWAS) to analyse over 500,000 single-nucleotide polymorphisms (SNPs) in samples from 589 cases of HL and 5,199 controls [45]. They reported exceptionally strong links with *HLA-DRA* at 6p21.32 with an odds ratio (OR) of 1.7 (95% confidence interval (CI) 1.58–1.72) (p = 2.84 × 10^−50^), but also with proto-oncogene *REL* at 2p16.1, encoding a transcription factor, two SNPs in *PVT1* at 8q24.21, encoding a long non-coding RNA, and two more in *GATA3* at 10p14, also encoding a transcription factor. A further GWAS reported a strong *HLA-DRA* link with a similar odds ratio: one reason for this may be that aberrant major histocompatibility loci enable malignant cells to evade immune recognition [46, 47]. Other researchers reported that variants of *TCF3* at 19p13.3, which code for transcription factor 3, influence the risk of developing HL, thus potentially aiding in diagnosis and management [48].

The mechanisms by which these (and other) genes contribute to the ability of malignant HL cells to evade recognition and destruction by the immune system include altered expression of programmed cell death protein 1 (PD-1) and its ligands (PD-L1 and PD-L2), a permanently active *JAK/STAT* pathway (Figure 1), and the avoidance of apoptosis via the PI3K/AKT/mTOR axis [47, 49]. The importance of genetic profiling for PD-1 (CD279, coded at 2q37.2 by *PDCD1* and expressed ubiquitously on Reed-Sternberg cells) is demonstrated by the positive effect of its blockade by nivolumab, in conjunction with standard chemotherapy, in advanced-stage classical HL [50]. Advances in liquid biopsies with PCR and NGS methods (often on blood samples, reducing the need to sample solid tissues such as those of the tumour) for circulating tumour DNA have facilitated lymphoma genotyping and the detection of mutations in *GNA13*, *ITPKB*, *SOCS1*, *STAT6*, and *TNFAIP3*, the latter coding for a molecule that can inhibit apoptosis, thus permitting the survival of malignant cells that would otherwise be marked for elimination [51].

An important document for practitioners in England is the National Genomic Test Directory for England, which lists genes that are linked to defined conditions that may be worthy of investigation [52]. However, unlike for NHL, the document has nothing specific to HL to offer. Wales, Scotland and Northern Ireland have their own documents [53–55].

### Non-Hodgkin Lymphoma (NHL)

Essentially a diagnosis of exclusion, meaning a failure to identify Reed-Sternberg cells, NHL has many variants that can be defined in the laboratory or clinically. These include histological intra-node anatomy (follicular/mantle/diffuse), cell morphology (large/small), CD-marker immunohistochemistry for particular lymphocyte subsets (/B/T/NK), and the location of the malignancy within the body (splenic/mucosal).

The ONS classified 4,555 deaths from NHL in England and Wales in 2023 as follicular (290 deaths), non-follicular (1,454 deaths), and mature T/NK-cell (311 deaths). However, the largest group was “other and unspecified types” (2,487 deaths) [1]. It is unclear whether this latter group genuinely represents lymphomas that are difficult to classify and/or those for which the pathological basis has not been reported. Despite the emphasis on death, these figures do not provide information on the cure rate of NHL: in the United States, the 5-year survival rate for NHL in the period 2019–2023 was 75.2% [56].

Further testament to the complexity of NHL is to be found in the WHO classification of lymphoid tumours, which includes HL, T and NK cell lymphomas, and plasma cell neoplasms, the latter to be discussed below [25] (Tables 3, 4). One of the best-known malignancies in this group is Burkitt lymphoma, as it was one of the first neoplasms to be linked to a specific infectious agent, i.e., EBV [15]. The principal genetic features of the majority of NHLs involve *MYC*, *BCL2,* and immunoglobulin genes at the *IgH* locus, leading to the following translocations: t(8;10)(q24;q32) (the most common), t(2;8)(p12;q24), t(8;22)(q24;q11), and t(14;18)(q32;q21), while t(8;14)(q24;q32) is also often present. Some cases have been linked to the *TP53* and *RB1* oncogenes, along with *ID3*, which codes for a DNA-binding protein that inhibits transcription [57, 58]. Alterations in other genes, such as the mutated *CCND3* gene at 6p21.1 (linked to deregulation of the cell cycle and so promotion of a malignant clone), may be present in ∼30% of cases [59–61]. Investigation of suspected cases uses an NGS panel and fluorescence *in situ* hybridisation (FISH)/PCR, focusing on translocations and rearrangements in the proto-oncogenes *MYC, BCL2* and *BCL6*, in addition to 11q abnormalities [52]. This is important because gains in 11q (the location of *CCND1*), or translocations such as t(11;14)(q13;q32), are likely to lead to the overexpression of the cell cycle regulator cyclin D1, thereby promoting lymphomagenesis [61]. Abnormalities in *CCND1* are present in many other cancers.

Large B-cell lymphomas (LBCL) are an extensive group with numerous subtypes, such as diffuse large B-cell lymphoma (DLBCL), the most common, accounting for between 25% and 50% of cases, depending on geography, and follicular lymphoma (22%). Other subtypes include high-grade tumours, those with both diffuse and high-grade features, tumours with an immune-privileged site (such as the eye), tumours related to fluid overload, and mediastinal grey zone lymphomas, a term often used to describe a lymphoma that is not easily classifiable. In many cases, EBV infection is an important aetiological factor [61–64]. As with other lymphomas, the leading genetic lesions in this group include *MYC, BCL2*, and *CCND1*, along with other oncogenes such as *ALK* at 2p23.2–23.1, which encodes CD246, the anaplastic lymphoma kinase, and *IRF4* at 6p25.3, which codes for the interferon regulatory factor 4 [62, 65]. In addition to heavy and light chain gene rearrangements, other genes of interest in B-cell NHLs include *EZH2* and *PLCG2* [65, 66]. Table 6 summarises the leading genetic abnormalities in the common NHLs.

**TABLE 6:** Molecular pathology of selected NHLs.

Lymphoma	Genetics
Diffuse large B-cell lymphoma	*BCL2* at 18q21.33, encoding an apoptosis regulator *BCL6* at 3q27.3, encoding a transcription regulator *CD79A/B* at 19q13.2 and 17q23.3, encoding parts of the B cell receptor *CREBBP* at 16p13.3, encoding a cAMP response-element-binding protein *EZH2* at 7q36.1, encoding a methyltransferase *MYC* at 8q24.21, encoding a nuclear phosphoprotein *MYD88* at 3p22.2, encoding myeloid differentiation primary response 88 and involved in signal transduction *PAX5* at 9p13.2, encoding a transcription factor
Follicular lymphoma	*ARID1A* at 1p36.11, encoding a molecule participating in transcription *DTX1* at 12q24.13, encoding a notch pathway regulator *EP300* at 22q13.2, encoding a histone acetyltransferase *MEF2B* at 19p13.11, encoding a transcription factor *CARD11* at 7p22.2, encoding a member of the caspase family *FOXO1* at 13q14.11, encoding a transcription factor *EZH2* and *CREBBP* (see above)
Primary mediastinal B-cell lymphoma	*CD274* at 9p24.1, encoding programmed death-ligand 1 *PDCD1LG2* at 9p24.1, coding for programmed cell death 1 ligand 2, CD273 *REL* at 2p16.1, encoding an NFκB-related transcription factor
Mantle zone lymphoma	Tumour suppressor *TP53* at 17p13.1, encoding p53 An *IGH::CCND1* translocation, i.e., t(11; 14)(q13; q32) *BTK* at Xq21.3–22, encoding Bruton’s tyrosine kinase *NOTCH1/2* at 9q34 and 1p13-11, encoding transmembrane receptors
Mucosal-associated lymphoid tissues	*MALT1* at 18q21.32, encoding the mucosa-associated lymphoid tissue lymphoma translocation protein 1, with a role in NFκB activation *MALT1* translocations t(11; 18)(q21; q21) with *BIRC3* at 11q22.2, encoding an inhibitor of apoptosis, t(14; 18)(q32; q21) with *IGH*, and t(1; 14)(p22; q32) between *IGH* and *BCL10* at 1p22.3, encoding another molecule involved in mitosis *FOXP1* at 3p13, encoding a transcription factor
*ALK-positive* large cell lymphoma	*ALK* at 2p23.2–23.1, encoding CD246 (anaplastic lymphoma kinase, hence ALK), a cell membrane receptor whose ligand is unknown *ALK* translocations with *CLTC* at 17q23.1, encoding for a clathrin, a component of organelle membranes, hence t(2; 17)(p23; q23), and with *NPM1* at 5q35.1, encoding for nucleophosmin, a molecule that binds nucleic acids, hence t(2; 5)(p23; q35) Large cell lymphomas with an anaplastic phenotype (i.e. are poorly differentiated with no clear mature phenotype) can be tested as above, except that investigation of *CLTC* is not required
*ALK-*negative large cell lymphoma	*DUSP22* at 6p25.3, encoding a phosphatase Tumour suppressor *TP53* (as above) *IRF4* at 6p25.3, encoding interferon regulatory factor 4, involved in signal transduction
Paediatric-type follicular lymphoma	*MAP2K1* at 15q22.31, encoding a signal-transduction mitogen-activated kinase

Tomacinschii and colleagues reviewed the role of NGS analysis in the NHLs [67]. Of particular importance from a clinical perspective is *BTK* (at Xq22.1), as it codes for Bruton’s tyrosine kinase, an enzyme with a role in B-cell signal transduction, and the target for a series of inhibiting drugs such as ibrutinib, which are effective in certain lymphomas and other B-cell malignancies [68]. Additional genes may be investigated in a lymphoma-specific or semi-specific manner [52, 67] (Table 6), while an extended GWAS [69] identified genes and loci of certain other lymphoma subtypes:

- DLBCL: *EXOC2* at 6p25.3, an HLA-B locus at 6p21.33, *NCOA1* at 2p23, and two independent SNPs in *PVT1* at 8q24.21.
- Follicular lymphoma: A locus near *CXCR5* at 11q23.3, another near *ETS1* at 11q24.3, *LPP* at 3q27.3–28, *BCL2,* and *PVT1* at 8q24.21.
- Risk of chronic lymphocytic leukaemia/small lymphocytic lymphoma linked to *ACTA2* at 10q23.31, *BCL2*, chromosome 11 open reading frame 21 at 11p15.5, *LEF1* at 4q25, *CASP10* or *CASP8* at 2q33.1 (coding for apoptosis enzymes), *CDKN2B-AS1* at 9p21.3, *PMAIP1* at 18q21.32, *BMF* at 15q15.1, QPCT at 2p22.2, and *ACOXL* at 2q13.
- Mantle zone lymphoma: *BTNL2* at 6p21.23, *BTK*, and a locus within *HLA-B.*

The importance of a precise genetic definition of lymphoma can be demonstrated in cases of DLBCL, where the main alteration is linked to clinical outcome [70]:

- Bad prognosis: altered *BTG1, CD58, CD79B, MYD88, NOTCH1, PIM1, PRMD1.*
- Intermediate prognosis: Aneuploidy*,* rearrangement in *BCL6, BCL10, CCND3, CD70, DTX1, NOTCH2, SPEN, TNFAIP3, TP53, UBE2A.*
- Good prognosis: altered *BRAF, BCL2, CD83, CREBBP, EP300, EZH2, IRF8, KMT2D,* rearrangement in *MYC, NFKBIE, SOCS1, SGK1 & TET2, STAT3, TNFSR14.*

### T and NK Cell Neoplasms

As with the B-cell lymphomas, there are numerous T/NK variants, with links to T-cell leukaemias [25]. Overall, the most common T-cell lymphomas comprise ∼10% of NHL diagnoses, the majority of them (∼6%) being peripheral, and others being cutaneous [62]. The investigation of T-cell tumours first focuses on rearrangements in the T-cell receptor (TcR), which is coded for by *TRA* (at 14q11.2)*, TRB* (at 7q34)*, TCD* (at 14q11.2) and *TRG* (at 7p14), coding for the alpha, beta, delta and gamma chains, respectively, generally by FISH and an NGS panel, as per the guidelines of the National Genomic Test Directory [52]. Other genes linked to the TcR include *PLCG1* at 20q12, the *VAV1* proto-oncogene at 19p13.3, *RHOA* at 3p21.31, and *CSNK1A1* at 5q32 [71]. Some of these genes may be clinically useful, be present in subtypes of lymphoma, and may also be present in an NGS panel [70, 72–74].

The kinase-coding oncogene *ALK* is of particular interest in anaplastic large cell lymphoma as it may be a translocation partner with many other genes, such as *NPM1*. It is also linked to breast-implant lymphoma, in which *STAT3, JAK1, JAK3, DNMT3,* and *TP53* may be involved [74, 75]. Yamagishi summarised the epigenetics of T-cell lymphomas, focusing on histone modifiers such as *EZH2* and *HDAC,* and methylation regulators such as *DNMT3A* and *IDH2* [76]. Table 7 summarises the genetics of T cell lymphomas, but with regard to NK cell lymphomas, the literature generally describes them in terms of T/NK cell lymphomas. As with B-cell lymphomas, the expression of these genes may be specific, semi-specific, or non-specific to a particular form of the disease.

**TABLE 7:** Genes in T-cell lymphomas.

Lymphoma	Genes
Follicular helper T-cell lymphoma	*IDH2, RHOA, TET2, DNMT3A, VAV1, CD28, ICOS, FYN*
Anaplastic large cell lymphoma	*ALK* (positive or negative), *DUSP22, JAK1, JAK3, NOTCH1, STAT3*
Peripheral T-cell lymphoma	*TET1, TET3, DNMT3A, TP53, PRDM1, CDKN2A/B, RB1* and *PTEN* loss, *STAT3* and *MYC* gain
Primary nodal EBV-positive (T/NK)	*TET2, PI3KCD, STAT3, TP53, CARD11*
Cutaneous T-cell lymphoma	*PLCG1, NFATC2, NFAT5, ZEB1, PRKCQ, RHOA, VAV1, PREX2, CTCF, ARID1A, TRRAP*
Hepatosplenic T-cell lymphoma	*STAT5B, STAT3, PIK3CD, SETD2, IN080, ARID1*; loss of 7p, amplification of 7q
Nasal NK/T-cell lymphoma	*TP53, DDX3X*, Del(6q), *STAT3, JAK3, STAT5B, BCOR*
T-cell lymphoblastic lymphoma	*NOTCH1/FBXW7, PTEN, RAS, KMT2D*
Gastrointestinal lymphoma	*JAK1, STAT3, TNFAIP3, KMT2D, TET2, SETD2, STAT5B, JAK3, GNAI2, TP53, MYC*

### Plasma Cell Neoplasms and Other Diseases With Paraproteins

This final group of WHO-defined mature B-cell neoplasms, notably myeloma [25], is discussed in a subsequent section.

### Non-Coding RNAs in Lymphoma

As with many other cancers, including those of the blood, the presence of various types of major non-coding RNAs (ncRNAs), including small interfering RNAs (siRNAs), circular RNAs (circRNAs), microRNAs (miRNAs), long non-coding RNAs (lncRNAs), Piwi-interacting RNAs (piRNAs) and others, has been reported in lymphoma [77, 78]. One of the earlier variants was the discovery of an intronic small nucleolar RNA; another was miRNA [79, 80]. Many types of ncRNAs regulate each other and mRNAs, often acting as “sponges” (in that they bind to and so nullify other RNAs), with abnormal levels having the potential to influence oncogene and tumour suppressor mRNA, and so carcinogenesis [77–80].

#### Hodgkin Lymphoma

Cordeiro et al. summarised reports of miRNAs in HL lymph nodes, cell lines, and microdissected Reed-Sternberg cells, listing many types, although only miR-21 was common to all three analyses [81]. They also described the potential roles for lncRNAs, such as MALAT1, FLJ42351, LINC00116, and LINC00461, and piRNAs, such as piR-651, piR-20365, and piR-20582. Paczkowska and Giefing updated and confirmed many of the miRNA findings, reporting RT-qPCR and small RNA-Seq methods that pointed to upregulation of let-7f, miR-9, miR-21, miR-23a, miR-27a, miR-155, miR-196a, along with downregulation of miR-138 and miR-150, with many other instances of up- and downregulation reported by single studies [82].

#### Non-Hodgkin Lymphoma

The heterologous nature of NHL is reflected by the many and different ncRNA profiles. In DLBCL, the upregulation of miR-155-5p and miR-21-5p has been consistently reported, with miR-155-5p and miR-222-3p linked to a poor prognosis [83–85]. Shi et al. reported altered expression of 21 miRNAs, which also point to links with pathophysiology [86]. For example, the downregulation of miR-26a, which targets p35 (CDK5R1), may interfere with the CDK5/STAT3 pathway, which is linked to cell proliferation and cell cycle progression [86]. Baghdadi and colleagues summarised the roles of various lncRNAs in different NHLs [78] (Table 8). Some of these lncRNAs, such as SNHG121, LINC0085, and SBFA2 in DLBCL, may also be useful in diagnosis and prognosis [84, 87].

**TABLE 8:** lncRNAs in non-Hodgkin lymphomas.

Lymphoma	Upregulated	Downregulated
Chronic lymphocytic leukaemia/small cell lymphoma	Lnc-IRF2-3, LEF1-AS1 lnc-ZNF667-AS1, treRNA, MIAT	GAS5, LincRNA-P21, BM&42401, DLEU2
Mantle cell lymphoma	HAGLROS, ROR1-AS1 MANCR, MALAT1	SNHG23
Burkitt lymphoma	MCM3AP-AS1, MINCR, NORAD	FAS-AS1
Follicular lymphoma	RP11-625L16.2	​
Diffuse large B-cell lymphoma	SNHG14, OR3A4, NEAT1 FIRRE, MALAT1, MALAT1, HOTAIR, LUNAR1, SMAD5-AS1, HULC	PANDA, FAS-AS1, lncRNA-21

#### T- and NK-Cell Lymphomas

As in B-cell lymphomas, there are numerous examples of abnormal miRNA expression in this variant: upregulation of miR-21, miR-155, miR-223 and miR-494, and downregulation of miR-15a, miR-16, miR-30b, miR-142, miR-146a, miR-148a, and miR-150 [88]. Other reports of deregulated lncRNAs include ZFAS1 overexpression (which may deregulate P53-mediated pathways), increased MALAT1 expression (linked to a poor prognosis), MTAAT associated with the progression of aggressive ALK-negative anaplastic large cell lymphoma, and MIR503HG promoting growth via the miR-503/Smuf2/TGFBR pathway [78, 88].

### Lymphoma and the Molecular Pathology Laboratory

The National Genomic Test Directory for England [52] has numerous entries for different forms of lymphoma. These can be investigated using combinations of methods that include complex variant detection, structural variant detection, copy number variant detection, and small variant detection using an NGS panel, FISH and whole genome screening (germline and tumour), although specific methods may apply in certain cases. Referrals include:

- B cell NHL: *IGH, IGK, IGL, EZH2, BTK,* and *PLCG*
- Burkitt lymphoma: *MYC, IGH::MYC, IGK::MYC, IGL::MYC, BCL2,* and *BCL6.*
- Burkitt-like lymphoma with 11q: 11q copy number variant detection
- Large B-cell-like lymphoma with *IRF4* rearrangement
- High-grade lymphoma: *MYC, BCL2* and *BCL6* rearrangement by FISH, t(8:14)(q34:q32) *IGH::MYC*, t(8:22)(q24:q11) *IGK::MYC*, t(2:8)(p12:q24) *IGL::MYC*, and t(14:18)(q32:q21) *IGH::BCL2* detected by FISH/RT-PCR, multi-target NGS panel for structural variants in *IGH::MYC, IGK::MYC, IGL::MYC, IGH::BCL2*, along with other rearrangements of *MYC, BCL2,* and *BCL6.*
- Primary mediastinal B-cell lymphoma: *CD274, PDCD1LG2,* and *REL* copy number by FISH.
- Mantle cell lymphoma: *TP53,* t(11;14)(q13;q32) *IGH::CCND1* by FISH/RT-PCR, *CCND1* rearrangement by FISH, and multi-target NGS panel for structural variants (*IGH::CCND1* and other *CCND1* rearrangements).
- Follicular lymphoma: t(14:18)(q32:q21) *IGH::BCL2* by FISH/RT-PCR, *BCL2* and *BCL6* rearrangement by FISH, multi-target NGS panel for small variants in *CARD11, CREBBP, EZH2, ARID1A, EP300, MEF2B,* and *FOXO1*, and multi-target NGS panel for structural variants in *IGH::BCL2, BCL2, BCL6,* and *MAP2K1* for the paediatric variant
- Intraocular lymphoma: *MYD88* hotspot
- MALT lymphoma: t(11;18)(q21;q21) *BIRC3::MALT1*, t(1;14)(p22;q32) *IGH::BCL10*, and t(14;18)(q32;q21) *IGH::MALT1* by FISH/RT-PCR, *MALT1, BCL10* and *FOXP1* rearrangement by FISH, multi-target NGS panel for structural variants in *BIRC3-MALT1, IGH-BCL10, IGH-MALT1* and other *MALT* rearrangements, *BCL10* rearrangements, and *FOXP1.*
- T-cell NHL: Multi-target NGS panel for small variants of *RHOA, DNMT3A, IDH2, and TET2*, TCR gene (*TRA, TRB, TRG, TRD)* rearrangement detection by multiplex sequencing or NGS.
- ALK-positive LBCL: *ALK* rearrangement, t(2;17)(p23;q23) *CLTC::ALK* and t(2;5)(p23;q35) *ALK::NPM1* by FISH/RT-PCR, and a multi-target NGS panel for structural variants (*CLTC::ALK, ALK::NPM1*, and other *ALK* rearrangements).
- ALK-Negative Anaplastic Large Cell Lymphoma (including primary cutaneous subtypes): *IRF4::DUSP22* and *TP63* rearrangements by FISH/RT-PCR, and a multi-target NGS panel for structural variants in *IRF4/DUSP22,* and *TP63.*
- ALK-positive Anaplastic Large Cell Lymphoma: t(2;5)(p23;q35) *ALK::NPM1* by FISH/RT-PCR, ALK rearrangement by FISH, and a multi-target NGS panel for structural variants of *ALK-NPM1* and other *ALK* rearrangements.
- NK Cell/Gamma-Delta T-Cell Lymphoma: Multi-target NGS panel for small variants in *STAT3* and *STAT5B.*
- Hepatosplenic T-Cell Lymphoma: Multi-target NGS panel for small variants in *STAT3* and *STAT5B*, i7q rearrangement by FISH, and a multi-target NGS panel for structural variants of i17q.
- Suspected lymphoma: Ig gene (*IGH, IGK,* and *IGL*) rearrangement detection by multiplex sequencing and rearrangement detection, NGS, and TCR gene (*TRA, TRB, TRG,* and *TRD*) rearrangement detection by multiplex sequencing and NGS. Karyotyping.

Despite intense scientific and clinical interest in hundreds of ncRNAs with pathophysiological, diagnostic and predictive values, they have yet to enter widespread laboratory practice. However, as siRNAs are becoming established as new therapeutic tools [89, 90], this may change.

### Summary of Lymphomas

Lymphoma is the most common blood cancer and has two forms, Hodgkin and non-Hodgkin, distinguishable by the presence of Reed-Sternberg cells. As the majority arise from malignant B lymphocytes, the main area of investigation is abnormalities in immunoglobulin genes (*IGH*, *IGK* and *IGL)*, which often form translocations with oncogenes such as *MYC* and *BCL2*. Of note, in Hodgkin lymphoma, there is a potential role for the programmed cell death protein (CD279) and its ligands, along with links with HLA types. Of the many forms of NHL, diffuse large B-cell lymphoma (DLBCL) is the most common, followed by follicular lymphoma. Alongside other forms, a large number of abnormal genes may be present. The importance of molecular genetics in DLBCL is that certain abnormalities are linked to clinical outcome. T cell lymphomas may be peripheral or cutaneous; investigations include determining abnormalities in genes coding for the T cell receptor. While many aberrant non-coding RNAs have been described in several lymphomas, they have yet to have a marked impact on diagnosis and management.

## Leukaemia

Table 2 shows the global and UK epidemiology of leukaemia. Of the subtypes of this cancer, myeloid leukaemia caused 2,686 deaths in this group in England and Wales in 2023; lymphoid leukaemia was linked to 1,206 deaths; monocytic leukaemia to 264 deaths; leukaemia of an unspecified cell type to 187 deaths; and other leukaemias of specified cell types caused 25 deaths [1]. Plasma cell leukaemia is discussed in the section on myeloma.

### Myeloid Leukaemia

An important document pertinent to this section is the WHO guidelines [91], which discuss myeloid leukaemia and other myeloid neoplasms, such as myelofibrosis, which will be covered in a separate section. However, a second relevant document is that of the International Consensus Classification [92], which addresses many of the same topics. Myeloid leukaemia may be classified as acute (in which case it is abbreviated to AML) and was linked to 2,356 deaths in England and Wales in 2023, while the chronic form (CML) was linked to 230 deaths, chronic myelomonocytic leukaemia to 253, acute promyelocytic leukaemia to 36, acute myelomonocytic leukaemia to 34 and acute monoblastic/monocytic leukaemia to 10 [1]. At this point, it is important to recognise that these data do not reflect incidence or prevalence; therefore, these deaths may, in some respects, be considered as failures of recognition or treatment.

#### Acute Myeloid Leukaemia (AML)

The dissection of the genetic pathogenesis of AML began in the 1970s, with reports of trisomy of chromosome 8 and a translocation between 9q and 22q (later shown to be the Philadelphia chromosome) [93–95]. This was subsequently extended to t(4;11)(q21;q23), t(8;21)(q22;q22), and a report on the activation of the *NRAS* proto-oncogene at 1p13.2, which encodes a GTPase with signal transduction pathway activity [96–98]. As the 1990s progressed, other reports appeared, such as that of inv(16) (p13;q22), which places *CBFB* at 16q22 (encoding a subunit of the core-binding factor) alongside *MYH11* at 16p13 (encoding myosin heavy chain), a fusion that forms an in-frame mRNA coding for a protein with potential transforming activity [99]. These and other findings led to the realisation that a classification based on genetics has advantages over one based on morphology, blast counts and surface marker expression [100]. This view gained credence in the current millennium as NGS methods expanded the number of identified mutations in AML to ∼400. Additionally, with its superior sensitivity and specificity, molecular genetics identified abnormalities in other genes, including *WT1* at 11p13 (encoding the Wilms tumour protein, a transcription factor)*, FLT3,* and *NPM1*, alongside fusion transcripts such as *MLL::MLLT3* and *DEK::NUP214* [101–103]. There was also a growing appreciation that the mutational status of certain genes, such as *NPM1, FLT3, NRAS,* and *CEBPA,* is linked to prognosis [104–107].

As knowledge increased, estimates of the frequency of mutations in various genes were published, the most common being *NPM1* (33%), t(15;17)(q22;q21) *PML::RARA* (13%) and *TP53* mutation or loss (8%) [108, 109] (Table 9). Additional analysis revealed many instances of co-mutation, an example being that of *NPM1*, where 50% of cases also exhibited a *DNMT3A* mutation*,* while 30% displayed an *FLT3-ITD* mutation [108]. Similarly, the number of translocations and inversions increased, with many involving genes already described above [110–112] (Table 10). Many genes can be grouped according to their function, for example, those involved in DNA methylation (*DNMTA3, IDH1/2* and *TET2)* or those acting as myeloid transcription factors (*CEBPA, ETV6, GATA2,* and *RUNX1)* [111].

**TABLE 9:** Frequency of leading gene mutations in AML.

Gene	Location	Product	Ref 107 frequency	Ref 108 frequency
*NPM1*	5q35.1	A nucleolar phosphoprotein that interacts with a ribosomal protein	30%–45%	27%
*DNMT3A*	2p23.3	DNA methyltransferase 3 alpha	34%	-
FLT3-ITD*	13q12.2	CD135, a tyrosine kinase cytokine receptor	28%–34%	20%–50%
FLT3-TKD*	13q12.2	CD135, a tyrosine kinase cytokine receptor	11%–14%	7%–10%
*IDH1*	2q34	Isoenzyme 1 of isocitrate dehydrogenase	4%–9%	4%–9%
*IDH2*	15q26.1	Isoenzyme 2 of isocitrate dehydrogenase	8%–19%	8%–19%
*TET2*	4q24	Methylcytosine dioxygenase 2	10%	-
*ASXL1*	20q11.21	Co-activator of the retinoic acid receptor	5%–16%	-
*CEBPA*	19q13.11	A transcription factor that binds the nucleotide sequence CCAAT	4%–9%	4%–9%
*KRAS*	12p12.1	A signal transduction GTPase	15%	-
*NRAS*	1p13.2	A signal transduction GTPase	25%	-
*KIT*	4q12	Receptor tyrosine kinase	20%–30%	2%
*KMT2A*	11q23.3	Lysine methyltransferase 2A	5%–10%	​
*RUNX1*	21q22.12	A subunit of transcription regulator core binding factor	5%–13%	4%–16%
*TP53*	17p13.1	p53, a tumour suppressor	5%–20%	8%–14%
*GATA2*	3q21.3	A haemopoietic transcription factor	-	9%
*TERT*	5p15.33	Telomerase reverse transcriptase	-	3%
*DDX41*	5q35.3	An RNA helicase	-	3%
*ETV6*	12p13.2	A transcription factor	-	1%

*ITD: Internal tandem duplication variant. *TDK: tyrosine kinase domain variant.

**TABLE 10:** Leading translocations and inversions in AML.

Abnormality	Genes fused
t(1; 22)(p13; q13)	*RBM15::MRTFA*
t(3; 5)(q25; q34)	*NPM1::MLF1*
t(5; 11)(q35; p15.5)	*NUP98::NSD1*
t(6; 9)(p22; q34)	*DEK::NUP214*
t(7; 12)(q36; p13)	*MNX1::ETV6*
t(8; 21)(q22; q22)	*RUNX1::RUNX1T1*
t(9; 11)(p21; q23)	*MLLT3::KMT2A*
t(9; 22)(q34; q11)	*BCR::ABL1*
inv(3)(q21; q26)	*GATA2::MECOM*
inv(16)(p13.3q24.3)	*CBFA2T3::GLIS2*
inv(16)(p13.1q22)	*CBFB::MYH11*

These may be detected using an NGS, panel or individually via FISH, and/or RT-PCR.

Genes involved in translocations not described in Table 9 or elsewhere include *MYH11* at 16p13.11, encoding a smooth muscle myosin, proto-oncogene *DEK* at 6p22.3, encoding a DNA-binder, *NUP214* at 9q34.13, encoding nucleoporin 214*, RBM15* at 1p13.3, coding for an RNA binding protein, *MRTFA* at 22q13.1–13.2, encoding a transcription factor, *MECOM* at 3q26.2, encoding a transcription factor*, NUP98* at 11p15.4, encoding a nucleoporin, *MLF1* at 3q25.32, encoding a protein with multiple roles in haemopoietic lineage commitment, *NSD1* at 5q35.3, encoding a histone methyltransferase, *MNX1* at 7q36.3, encoding a transcription factor that binds regulatory elements, *MLLT3* at 9p21.2, encoding a molecule that increases the rate of RNA polymerase II-critical haemopoietic transcription factors [113]. The importance of this genetic analysis is demonstrated by its links with prognosis [112]:

- Favourable: t(8;21)(q22;q22.1)/*RUNX1::RUNX1T1*, inv(16)(p13;q22), t(16;16)(p13.1;q22)/*CBFB::MYH11*, mutated *NPM1* without *FLT3-ITD*, bZIP in-frame mutated *CEBPA*
- Intermediate: Mutated *NPM1* with *FLT3-ITD*, wild-type *NPM1* with *FLT3-ITD*, t(9;11)(p21.3;q23.3)/*MLLT3::KMT2A*, and cytogenetic and/or molecular abnormalities not classified as favourable or adverse
- Adverse: t(6;9)(p23;q34.1) *DEK::NUP214*, t(v;11)(v;q23.3); *KMT2A-*rearranged, t(9;22)(q34.1;q11.2) *BCR::ABL1*, t(8;16)(p11;p13) *KAT6A::CREBBP*, inv(3)(q21.3q26.2) or t(3;3)(q21.3;q26.2)/GATA2, MECOM (EVI1), t(3;v)(q26.2;v)/*MECOM* (EVI1) rearranged, −5 or del(5q); −7; −17/abn(17p), complex karyotype, monosomal karyotype, mutated *ASXL1, BCOR, EZH2, RUNX1, SF3B1, SRSF2, STAG2, U2AF1, ZRSR2,* or *TP53*

Furthermore, knowledge of certain mutations can inform treatment decisions. *IDH2* can be targeted with enasidenib, and *IDH1* with ivosidenib and olutasidenib (all of which inhibit the product of these genes, isocitrate dehydrogenase). *FLT3* can be targeted with midostaurin, gilteritinib, or quizartinib (each of which inhibits tyrosine kinase), while the BCL protein can be targeted with venetoclax [109, 110, 114, 115]. The National Genomic Test Directory for England for AML is extensive [52], reflecting the text described above, and many tests are also part of the directories of other UK nations [53–55]. Entries include a multi-target NGS panel for small variants in *NPM1, CEBPA, RUNX1, FLT3, IDH1, IDH2, KIT, WT1, ASXL1, SRSF2, STAG2, RAD21, TP53, KRAS, NRAS, KMT2A(MLL)-PTD, PPM1D, DDX41, PHF6,* and *CUX1*, and a multi-target NGS panel for structural variants to include the detection of t(15;17)(q24;q21) *PML::RARA*, t(8;21)(q22;q22) *RUNX1:RUNX1T1*, inv(16)(p13.1q22) *CBFB::MYH11*, t(9;11)(p21;q23) *MLLT3::KMT2A* and other 11q23 rearrangements, t(6;9)(p22;q34) *DEK::NUP214*, inv(3)(q21;q26) *GATA2::MECOM*, t(1;22)(p13;q13) *RBM15::MRTFA*, t(9;22)(q34;q11) *BCR::ABL1*, t(3;5)(q25;q34) *NPM1::MLF1*, t(5;11)(q35;p15.5) *NUP98::NSD1*, t(7;12)(q36;p13) *MNX1::ETV6*, inv(16)(p13.3q24.3) *CBFA2T3::GLIS2* and other *NUP98* rearrangements. Regarding MRD, *NPM1, PML::RARA, CBFB::MYH11, RUNX1:RUNX1T1,* and *BCR::ABL1* are described, with a testing method being RT-qPCR.

#### Acute Promyelocytic Leukaemia (APL)

APL was described in detail in 1957. A key point in the molecular pathology of this cancer can be traced back to the 1980s with reports of t(15;17)(q22;q21) [116–119], subsequently shown to bring together *PML* at 15q24.1 and *RARA* at 17q21.2 to form a PML-RARA fusion protein [120]. The UK’s National Institute for Health and Care Excellence (NICE) suggests that 98% of cases of APL are due to this mutation and that 10% of AML cases are APLs. If so, then approximately 259 cases in England alone would be expected [121]. With only approximately 30 deaths [1], this represents a considerable treatment success, as the use of all-*trans*-retinoic acid alongside idarubicin (which interferes with topoisomerase II) can induce a complete remission in the majority of patients. Accordingly, APL has been described as “the most curable form of acute leukaemia” [121, 122]. The remaining 2% of APL cases all involve *RARA*, but translocated with *ZBTB16, NPM1, NUMA1, STAT5B, PRKAR1A, FIP1L1, BCOR, NABP1, TBL1XR1, GTF2I, IRF2BP2* and *FNDC3B*, while almost half of all APL cases have an abnormal karyotype, primarily del(7q) and +8 [123].

#### Chronic Myeloid Leukaemia (CML)

The molecular genetics of CML have much in common with APL, in that, unlike AML, they are almost always linked to a single genetic defect. As previously mentioned [8, 9, 14, 18], this is the presence of t(9;22)(q34;q11), which results in the oncogene *BCR::ABL1*. This codes for a 210 kDa fusion protein (BCR-ABL1) with tyrosine kinase function that activates second messengers such as JAK2 and STAT5, which is the basis of this particular form of carcinogenesis (Figure 1) [124, 125]. This kinase proved to be inhibitable, leading to one of the first targeted drug treatments for cancer: imatinib; however, in some patients (up to 15% in some studies) there was resistance to the drug [126, 127]. Fortunately, other tyrosine kinase inhibitors (nilotinib and dasatinib, both markedly more effective than imatinib) that target other parts of the fusion protein were developed to address this issue, and others have been subsequently added to this list [125, 128–130]. Unsurprisingly, *BCR::ABL1* is the lesion of choice for the detection of MRD, with Salmon and colleagues discussing methods and transcript type [131].

Reflecting the small number of genetic lesions in CML, entries for CML in the National Genomic Test Directory for England focus on *BCR::ABL1* by multiplex analysis, FISH and RT-qPCR [52]. Karyotyping may be used for the detection of +8, +19, −7/7q, −5/5q, i(17q), 12p copy number, and t(9;22)(q34;q11) *BCR::ABL1* (including p190 and p210 variants), with FISH used for *MECOM* and 11q23 (*KMT2A*) rearrangement. Several of these may also be part of an NGS panel [52].

### Lymphoid Leukaemia

Data from the ONS [1] shows the leading form of lymphoid leukaemia (causing 1,206 deaths in England and Wales in 2023) to be chronic lymphoid leukaemia (CLL), with 877 deaths (72.7%), with the acute variant linked to 214 deaths (17.7%) in the same year. Less common forms include hairy-cell leukaemia (33 deaths, 2.7%) and prolymphocytic leukaemia (31 deaths, 2.6%).

#### Chronic Lymphocytic Leukaemia (CLL)

This form of leukaemia is inevitably of B-cells, and, reflecting their frequently close aetiopathogenesis, the WHO guidelines describe CLL and small lymphocytic lymphoma together as mature B-cell neoplasms [25]. The familial nature of CLL, implying genetics, can be traced back to the 1960s, with discoveries of abnormal karyotypes, translocations and rearrangements in immunoglobulin genes emerging in the 1980s [132–137]. In the current millennium, reviews pointed to frequencies of del(13q14) at ∼50%, del(11q22–23) at 18%–20%, +12 at 12%–15%, del(6q21) at 10%, and del(17p13) at 5%–10% [138, 139]. These deletions are important because the absence of *TP53* (a tumour suppressor located at 17p13.1), *DLEU7* (*located* at 13q14.3, an inhibitor of transcription factors NF-*k*B and NFAT), and *ATM* (located at 11q22.3, required for damaged DNA repair) promotes leukaemogenesis [140, 141].

NGS techniques have subsequently shown loss-of-function mutations in *NOTCH1* (located at 9q34.3 and coding for a membrane receptor with epidermal growth factor-like repeats) and *SF3B1* (located at 2q33.1 and encoding a splicing factor) in CLL [142, 143]. In one study, mutations in *NOTCH1* were present in 21.8% of cases, in *SF3B1* in 12.6%, in *ATM* in 11.1%, in *TP53* in 10.6%, and in *BIRC3* in 4.2% of cases [144]. These, and other genes, such as immunoglobulin genes and *BTK* genes, are frequently included in lists of diagnostic and prognostic genes. For example, while del(13q14), with a prevalence of 50%–60% at diagnosis, is indicative of a prognostic risk, the presence of del(11q22-23) (present in 5%–20% of cases at diagnosis) and del(17p13) (present in 1%–13% of initial diagnoses) are both associated with a poor prognosis [145, 146]. Other mutated genes, present in 1.8%–6.3% of cases, are shown in Table 11 [143]. Several risk loci have been identified by GWAS, such as 6p21.3, the nearest genes being *BAKI* 3 and *IRF4*, both of which are close to 6q24-25, and *IRF8*, the latter two genes coding for interferon regulatory factors [147]. MRD in CLL can be detected by multi-colour fluorescence flow cytometry to analyse CD markers relevant to the tumour, although the preferred molecular method is to analyse immunoglobulin gene rearrangements using NGS [148, 149].

**TABLE 11:** Genes linked to CLL with low frequency.

Gene	Location	Product
*BIRC3*	11q22.2	A molecule that contributes to the inhibition of apoptosis
*CHD1B*	15q26.1	A helicase
*DDX3X*	Xp11.4	A helicase
*FBXW7*	4q31.3	A molecule that contributes to the ubiquitin protein ligase
*KLHL6*	3q27.1	A molecule involved in B-lymphocyte antigen receptor signalling and germinal centre formation
*LRP1B*	2q22.1–22.2	The low-density lipoprotein receptor-related protein
*MAPK1*	22q11.22	Mitogen-activated kinase-1
*MYD88*	3p22.2	A transduction adaptor
*PLEKHG5*	1p36.31	A protein that interacts with GDP/GTP
*POT1*	7q31.33	A molecule that is part of shelterin, which protects telomeres
*TGM7*	15q15.2	A transglutaminase
*XPO1*	2p15	Exportin, with a role in exporting proteins and RNAs from the nucleus

Addressing the above, the National Genomic Test Directory for England [52] refers to multi-target NGS panels for small variants in *TP53, BTK, PLCG2,* and *BCL2*; for copy number variants in *TP53, ATM, DLEU2/7, RBI,* and +12; and for *IGH, IGK,* and *IGL* rearrangement and hypermutation detection. FISH may be the technique of choice for abnormalities in *TP53*, 11q, 13q, and del(17p). If present, these may guide treatment, such as the use of idelalisib, an inhibitor of phosphoinositide 3-kinase, in patients with del(17p) or a *TP53* mutation [150], and ibrutinib, an inhibitor of Bruton’s tyrosine kinase [151].

#### Acute Lymphoblastic Leukaemia (ALL)

Early chromosomal studies of this disease reported abnormalities such as t(21;22), t(8;14), t(9;22), t(4;11), t(11;14), t(1;3) and (t(9;22)(q34:q11)/*BCR::ABL1*), also known as the Philadelphia chromosome [152–156]. Others helped to differentiate the sublineages of the malignancy. In B-cell ALL, these include t(8;14)(q24;q32)/*MYC::IGH*, t(2;8)(p11;q24)*/MYC::IGK*, t(8;22)(q24;q11)/*MYC::IGL*, t(11;14)(q13;q32)/*BCL1::IGH,* and t(14;18)(q21;q32)/*BCL2::IGH*. In T-cell diseases these include t(8;14)(q24;q11)/*MYC::TCR*, t(11;14)(p15;q11)/*TTG1::RHOM1*, t(7:11)(q35;p13)*/TTG2::RHOM2*, t(11;14)(p13;q32)/*TCL2::TCR,* and inv(14)(q11;q32)/*TCL1::IGH* [157, 158].

More recent reports recognised many of these abnormalities and described additional chromosomal (hypo- and hyperploidy, iAMP21) and genetic lesions, some of which are rearrangements and indels, while others are translocations (Tables 12, 13) [25, 28, 159–165]. There are many examples of co-mutations, some of which are statistically significant and may therefore have pathogenic implications. For example, in a study of 804 ALL patients, an *IKZF1* deletion at 7p12.2*,* which encodes a zinc finger transcription factor, was linked to a *BCR::ABL1* fusion, whereas it was inversely associated with an *RTV6::RUNX1* fusion, with both showing a strong probability of p < 0.001, implying pathogenic significance [159]. Elsewhere, it was reported that 35% of Ph-like B-ALLs have *PAX5* alterations [160]. Notably, *KMT2A* has at least six translocation partners–with *ELL,* forming t(11;19)(q23;p13.1), with *AFDN*, forming t(6;11)(q27;q23), with *AFF1,* forming t(4;11)(q21;q23), with *MLLT1*, forming t(11;19)(q23;p13.3), with *MLLT3*, forming t(9;11)(p21;q23), and with *MLLT10*, forming t(10;11)(p12;q23).

**TABLE 12:** Gene mutations in ALL.

Gene	Location	Product
*IKZF1*	7p12.2	A zinc-finger transcription factor
*PAX5*	9p13.2	B-cell lineage-specific activator
*NOTCH1*	9q34.3	A membrane receptor with epidermal growth factor-like repeats
*FBXW7*	4q31.3	A transcription factor linked to ubiquitin
*KMT2A*	11q23.3	A histone methyltransferase
*ETV6*	12p13.2	A transcription factor
*TCF3*	19p13.3	A transcription factor
*PBX1*	1q23.3	Pre-B-cell leukaemia transcription factor
*CSF1R*	5q32	Colony-stimulating factor 1 receptor
*PDGFRB*	5q32	Platelet-derived growth factor receptor-β
*HGF*	7q21.11	Hepatocyte growth factor
*DUX4*	4q35.2	A transcription activator
*ZNF384*	12p13.31	A zinc-finger transcription factor
*MEF2D*	1q22	Myocyte enhancer factor 2D
*NUTM1*	15q13	A regulator of proliferation
*EBF1*	5q33.3	Regulates proteins needed for B-cell differentiation
*TAL1*	1p33	A transcription factor
*TLX1*	10q24.31	A transcription factor

**TABLE 13:** Leading translocations in ALL.

Translocation	Genes fused
t(9;22)(q34:q11)	*BCR::ABL1*
t(12;21)(p13;q22)	*ETV6::RUNX1*
t(1;19)(q23;p13)	*TCF3::PBX1*
t(5;14)(q31.1;q32.1)	*IGH::IL3*
t(17;19)(q22;p13)	*TCF3::HLF*
t(5;14)(q35;q32.2)	*BCL11B::TLX3*
t(1;7)(p32;q11)	*TRB:TAL1*
t(1;14)(p32;q11)	*TRA::TAL11*
t(10;14)(q24;q11)	*TLX1::TRD*

These may be detected using an NGS, panel or individually via FISH, and/or RT-PCR.

Genetic lesions linked to a poor outcome include *KMT2A* fusions, near-haploidy (24–30 chromosomes), low hypodiploidy (31–39 chromosomes), iAMP21, *TCF3::HLF*, *CDKN2A/B* deletions, and *ABL*-class fusions. CNS disease at diagnosis is also associated with a poor prognosis [166, 167]. As with many other malignancies where there is a known genetic lesion, such as *BCR::ABL1*, this will be used for MRD. An alternative is immunoglobulin and T-cell receptor gene rearrangements, while other candidates include *ETV6::RUNX1* and *IKZF1* deletion [167, 168].

The National Genomic Test Directory for England regarding ALL is extensive and cannot be fully reproduced here, although many of the genes discussed above are referenced, and there is considerable duplication [52]. However, whole genome sequencing (WGS) for all variant types in the germline and tumours is described, as are global copy number changes by FISH for hyperdiploidy, high hyperdiploidy, near haploidy, and low hypodiploidy. These, and copy number changes of *IKZF1*, *CDKN2A, CDKN2B, BTG1, EBF1, PAX5, RB1, PAR1* region (*CRLF2, CSF2RA,* and *IL3RA*), and *ETV6,* can also be detected by a multi-target NGS panel.

As with many other cancers, patients starting on thiopurine-based chemotherapy will be tested for *TPMT* and *NUDT15* variants using SNP/small variant detection, as the enzyme variants coded for by these genes affect the biological activity of the drugs: “weak” acting enzyme isoforms can lead to prolonged cytotoxic effects, which may have adverse clinical consequences, while “strong” isoforms may render the drugs less effective.

MRD can be detected using QF-PCR for t(9;22)(q34:q11) *BCR::ABL1*, t(12;21)(p13;q22) *ETV6::RUNX1*, t(1;19)(q23;p13)/*TCF3::PBX1*, t(17;19)(q22;p13)/*TCF3::HLF*, t(4;11)(q21;q23)/KMT2A::AFF1, t(11;19)(q23;p13.3)/KMT2A::MLLT1, t(6;11)(q27;q23)/*KMT2A::AFDN*, and t(9;11)(p21;q23)/*KMT2A::MLLT3*, and for complex variation in *IGH, IGK, IGL, TRA, TRB, TRG*, and *TCRD*. Copy number variation (CNV) detection at a genome-wide level using FISH can be used to identify del(1p33), the location of *TSL1*, *iAMP21* and the location of *RUNX1*.

#### Hairy Cell Leukaemia (HCL)

The WHO guidelines place this condition within a small group of splenic B-cell lymphomas and leukaemias [25]. The principal morphological feature of this disease, the “lace-like” cytoplasmic extrusions, was described in 1958 and led to the condition being named “hairy cell leukaemia” in 1966 [169, 170]. Probing of HCL cells from 48 patients revealed that they all had a V600E (valine > glutamic acid) mutation in *BRAF* at 7q34, encoding B-Raf, a serine/threonine kinase, and was subsequently defined as a proto-oncogene [171]. *BRAF* mutations have been reported in several other cancers, and, such as the BCR-ABL1 fusion protein, the protein product is a target for inhibitors such as dabrafenib, encorafenib, ibrutinib, and vemurafenib [172, 173]. The National Genomic Test Directory for England [52] describes a multi-target NGS panel for small variants in *BRAF*, the V600 hotspot, immunoglobulin heavy chain (*IgH*) rearrangements, and small variants in *MAP2K1* at 15q22.31, with mutations present in a third of cases that are unmutated for *BRAF* ^*V600E*^ [52, 173].

#### Monocytic Leukaemia

This malignancy was linked to 6% of all leukaemia deaths in England and Wales in 2023 [1], placing it third after myeloid and lymphoid leukaemias, reflecting its frequency in normal full blood counts. First described in detail in 1928 and further elaborated upon in 1975, a monoblastic variant was reported in 1980, with definitions based on morphology, cytochemistry and CD markers, leading to the French-American-British (FAB) system, in which monocytic leukaemia is designated as AML type M5 [174–176]. The WHO guidelines place it with variants of AML. Many commentators have pointed to interrelationships between the different forms of leukaemia depending on differentiation [67, 177]. He and colleagues emphasised the value of *LILRB4* and *LRRC25* protein products as biomarkers in the monocytic form, while others suggested the value of *KMT2A* (described above) and the internal tandem duplication variant of *FLT3* at 13q12.2, coding for the Fms-related receptor tyrosine kinase 3, in paediatric disease. Additionally, they highlighted the importance of the cell adhesion molecule 1 encoded by *CADM1* (formerly *IGSF4*) at 11q23.3 [178–180].

#### Myelomonocytic Leukaemia (MML)

As the name suggests, this variant, first described in detail by Osgood in 1968, occupies the space between myelocytic and monocytic leukaemia, defined by the FAB system as M4 [181, 182]. Bower and colleagues were among the first to report genetic changes in *MML* (now *KMT2A*) and t(6;11)(q27;q23), while Levine et al. described the mutant *JAK2* ^*V617F*^ in 9 out of 116 (7.8%) cases of chronic MML [183, 184]. As array-CGH and NGS have become more widely used, the list of genetic abnormalities has been expanded, with several more cytogenetic abnormalities and mutations reported (Table 14) [185, 186], alongside mutations in numerous genes in the spliceosome component pathway such as *SF3B1, SRSF2, U2AF1 (U2AF35), ZRSR2, SF3A1, PRPF40B, U2AF2 (U2AF65)* and *SF1* [187, 188]. Genes with a frequency of ≤10% include *NRAS, DNAH2, NPM1, IDH2, PTPN11, CSMD1, PTCH1, CDH23*, and *JAK2*, while in a study of 69 patients, 15 subjects (21.7%) had 6 mutations, 8 subjects (11.6%) had 8, and three sets of 5 patients had 5, 9 or 10 mutations. Unsurprisingly, the total number of mutations had a strong impact on the outcome. *ASXL1, NRAS, TET2, SRSF2, SETBP1*, and *RUNX1* status can be used to create a risk stratification model to predict outcomes that include transformation to AML, myelodysplasia, and death [188–191]. The National Genomic Test Directory for England does not refer to MML, but it does have a section on juvenile MML, to be discussed in a subsequent section, which cites many of the genes described above.

**TABLE 14:** Chromosomal and gene abnormalities in myelomonocytic leukaemia.

Cytogenetic abnormality	Frequency	Molecular mutation	Frequency
Any aberration	26.9%	*SRSF2*	50%
−7	6.3%	*TET2*	45%
+8	6.2%	*ASXL1*	40%
+21	1.5%	*RUNX1*	24%
del(5q)	1.5%	*RAS*	22%
Iso(17)	1.5%	*NRAS*	18%
del(12p)	1.0%	*SETBP1*	15%
el(20q)	0.8%	*FAT4*	14%
​	​	*KRAS*	13%
​	​	*CBL*	13%
​	​	*ARIH1*	12%
​	​	*EZH2*	11%

From references [185–190].

#### NK Cell Leukaemia

In the 1980s, it was recognised that a small number of lymphocytes had phenotypes that were larger and more granular than “standard” lymphocytes, with many of these (∼85%) subsequently defined as NK (= natural killer) cells, which express CD56 but not CD3, the T-cell marker [192, 193]. Chromosomal abnormalities reported in the late 1990s included + X, +8, del(6q21-23), del(13q), del(17p) and del(17q), along with rearrangements in 11q23 [194]. Current WHO guidelines have a section on mature T-cell and NK-cell neoplasms [25], and within this list are NK-large granulocytic lymphocytic leukaemia and NK/T-cell lymphoma. The latter is characterised by mutations in *TP53, DDX3X, STAT3, JAK3, MGA, BCOR, ECSIT,* and *MCL1*, alongside deletions in chromosomes 6, 8 and 14 [195]. Aggressive NK cell leukaemia may harbour abnormalities in *STAT3* or *STAT5B (*present in approximately half of cases), which code for molecules of the JAK/STAT pathway, while array CGH has reported loss of 7p15.1-q22.3 and 17p13.1, with gains of 1q23.1-q23.2 and 1q31.3-q44 in NK leukaemia compared to extra-nodal NK lymphoma [196]. Others have used WGS to report mutations in *TP53* (present in 34% of cases), *TET2* (present in 28% of cases), *CREBBP*, and *MLL2* (both present in 21% of cases) in aggressive NK cell leukaemia [197]. The National Genomic Test Directory for England points to multi-target NGS panels for detecting small variants of *STAT3* and *STAT5B* in NK cell/gamma-delta T-cell lymphoma, and for investigating large granular lymphocyte leukaemia [52].

#### Other Leukaemias

Chronic neutrophilic leukaemia (CNL), in which the blood film is primarily dominated by mature neutrophils and often a small number of metamyelocytes, was described over a hundred years ago as hyperleukocytosis [198]. Diagnosis first relies on the elimination of differential diagnoses such as a leukaemoid reaction from CML or atypical CML, the latter of which is achieved by failure to find *BCR::ABL1* and rearrangement of *PDGFRA, PDRGRB,* or *FGFR1* [67, 199–201]. As with all very rare conditions, confidence in the diagnosis is limited by small numbers. A study of 9 patients with CNL found one individual to have *JAK* ^*V617F*^ and eight to have a *CSF3R* mutation; of these, five also had a *SETBP1* mutation [200], while a larger study of 39 patients found mutations in *ASXL1* (in 77%), *CSF3R* (62%), *SRSF2* (41%), *SETBP1* (40%), *TET2* (20%), and *U2AF1* (14%), with *NRAS, PTPN11, JAK2, CBL, ABL1, GNB1, USAF2,* and several others found at a frequency of <10%, with abnormal cytogenetics in over 30% of patients [201].

There are several differential diagnoses for eosinophilic leukaemia, such as variants of CML, ALL, myelodysplasia, and hypereosinophilic syndrome. Although there are no eosinophil-specific genetic lesions, rearrangements involving *PDGFRA, PDGFRB*, *FGFR1,* or *PCM1::JAK2* may be present [67, 202, 203]. Investigation of chronic basophilic leukaemia focuses on the *MYB::GATA1* fusion arising from t(X;6)(p11;q23), although there are reports of the Philadelphia chromosome t(9;22)(q34;q11), t(3;6)(q21;p21), and t(16;21)(p11;q22) [204]. The 2022 WHO guidelines refer to numerous forms of mastocytosis, with *KIT* variants (such as D816V) possibly linked to a systemic form, but there may also be a role for *TET2, SRSF2, ASXL1, RUNX1*, and *JAK2* [67, 205].

### Non-Coding RNAs in Leukaemia

#### Lymphoid Leukaemias

The potential pathological roles of miRNAs were first reported over 20 years ago, with one of the earliest studies showing that del(13q14), which is present in over 50% of CLLs, is the location of miR15 and miR16*.* This suggested a route to transformation via *BCL2-*directed apoptosis [206, 207]. Calin and colleagues subsequently reported that a 13-miRNA signature was linked to the time from the initial CLL diagnosis to the start of treatment [208], opening the door to its use in diagnosis and management [209]. More recently, several miRNAs have been linked to prognosis in CLL (Table 15) [210, 211].

**TABLE 15:** miRNAs in CLL.

Possible prognosis	Expression in CLL	miRNA
Good	Upregulated	miR-223, miR-29, miR-34, miR-145, miR-650
Bad	Upregulated	miR-155, miR-181 miR-17/92
Good	Downregulated	miR15a/miR-16–1, miR-9-3 mir-138^a^, miR-708^a^
Bad	Downregulated	miR-126, miR-3151 miR-9-3^b^, miR-143^b^

^a^ Bad when downregulated.

^b^ Good while upregulated.

There are numerous reports of altered miRNAs in ALL. For example, Alvarez-Zuniga and colleagues reported that plasma miR-511, miR-34a, miR-22, miR-26a, miR-221, and miR-223 all exhibit good sensitivity and specificity for B-cell progenitor ALL, although others found miR-92a and miR-638 to be less discriminatory, as did another research group with respect to miR-21, miR-24, miR-26, miR-133b, and miR-148a in peripheral blood mononuclear cells [212]. Mendiola-Soto et al. summarised miRNAs in ALL (Table 16), some of which, such as upregulated miR-137 and miR-510, and downregulated miR-100 and miR-151, are differentially expressed in T-ALL versus B-ALL [213], while others hypothesised roles for miRNAs in haemopoiesis and leukaemogenesis [213–215]. MiR-22, miR-24, miR-150, miR-148a, miR-155, miR-633, and others may play a role in HCL by potentially activating the MAP-JNK pathway and possibly forming a unique signature [216].

**TABLE 16:** miRNAs in ALL.

Upregulated
miR-7e, miR-9, miR-9*, miR-34a, miR-92a, miR-100, miR-125b-1, miR-128, miR-130b, miR-142-3p, miR-146a miR-155, miR-181, miR-181a [111,117], miR-181b [111,115], miR-210, miR-222, miR-339, miR-363, miR-511, miR-638, miR-1943, miR-1841, miR-1931, miR-198, miR-1890, miR-1902
Downregulated
let-7e, miR-18a, miR-26a, miR-30a, miR-100, miR-126, miR-143, miR-145, miR-196a, miR-196b, miR-199b-3p, miR-200c, miR-203, miR-221, miR-223, miR-326, miR-373*, miR-451, miR-582-5p, miR-1893, miR-1971*, miR-1834, miR-1842*, miR-1842

There is also a substantial body of literature on lncRNAs. Baghdadi and colleagues summarised the role of these molecules in lymphopoiesis, with possible roles in lineage differentiation [78]. Altered lncRNAs in B-cell ALL include LINC0098 (whose target is miR-330-5p) and ZEB1-AS1 (targeting the IL1/STAT3 pathway), which act as oncogenes, and LINC00221 (targeting miR-152-3P) and CASC15 (targeting *SOX4*)*,* which act as tumour suppressors [217].

#### Myeloid Leukaemias

One of the earliest comprehensive studies on miRNAs in AML described an expression signature (including miR-128a and −128b, let7b, and miR-223) that could be used to discriminate it from ALL, while another studied 26 species, finding miR-126, miR-130a, miR-93, miR-125a, and miR-146 to be downregulated, while high expression of miR-191 and miR-199a were predictors of poor survival [218, 219]. In addition, other studies reported 17 upregulated and 16 downregulated variants, and that cases with t(15;17) had a unique miRNA signature in 14q32 that included miR-127, miR-154, miR-299, miR-323, miR-368, and miR-370 [220]. More recently, Bhattacharya and Gutti summarised the roles of miRNAs such as miR-124, miR-126, miR-223, and miR-193b. Meanwhile, Fletcher and colleagues described 17 miRNAs with the potential to serve as therapeutic targets, such as mimics of miR-29b and miR-181a, which have been shown to be effective in cell biology and in animal models. Finally, Liu et al. reported that high levels of miR-362-5p and low levels of miR-34a are linked to a poor prognosis [221–223].

There is also a large body of literature on lncRNAs in AML, and, as with other ncRNAs, many interact with other ncRNAs [221]. Examples of this regulation include NEAT1, which is under-expressed in AML and targets miR-23a-3p, with possible consequences for increased myeloid cell proliferation and for apoptosis. Other examples include SATB1-AS1, which binds to and so acts as a neutralising sponge for the miR-580, and MALAT1, which sponges the miR-328-3p, itself acting on the cell cycle regulator *CCND2* [223, 224]. There are many examples of lncRNAs with a role in drug resistance, such as DANCR, which, by repressing miR-874-3p, raises ATGL16 protein levels (a key component of the autophagy mechanism) and confers resistance to cytarabine, while others, such as KCNQ1OT1, which is upregulated in AML, act on miR-296-5p and may have a role in *c-Myc* expression and in suppressing apoptosis [224–227]. Once data reached a critical mass, meta-analyses became possible, one of which, pooling four studies, showed that elevated miR-155 was linked to AML, with an odds ratio (95% confidence interval) of 1.68 (1.41–2.00) [228].

One of the first reports of an ncRNA in CML came from Venturi and colleagues, with evidence of the importance of the miR-17–92 polycistron at 13q31-32. This polycistron includes miR-17-5p, miR-17-3p, miR-18a, miR-19a, miR-20a, miR-19b, and miR-92–1 [228]. Subsequent reports have described the downregulation of miR-10a, miR-150 (which normally targets *MYB*), miR-328, and miR-181A, with the upregulation of miR-130A [229–231]. However, the key link to pathophysiology is that numerous miRNAs that would normally target *BCR::ABL1* are downregulated, with examples being miR-29b, miR-30a, miR-23a, and miR-342-5p, while the upregulation of others influences alternative pathways, such as miR-29a-3p, which decreases apoptosis, and miR-126-3p, which increases therapy resistance [232].

As in other blood cancers, lncRNAs may participate in the disease process. Examples include the downregulation of BGL3, normally acting as a tumour suppressor by targeting several miRNAs and thereby altering the function of *PTEN*, and the upregulation of lncRNA H19, which targets *c-MYC* when upregulated, resulting in disease progression and a poor prognosis. As with miRNAs, lncRNAs, such as HOTAIR and HULC, also play a role in resistance to therapy with tyrosine kinase inhibitors [232, 233].

A third type of ncRNA is the closed, circular RNA molecule known as circRNA, which is of interest in blood cancers [221, 223]. Many circRNAs may regulate miRNAs and consequently downstream genes, such as the circ-HIPK2-miR-124a-*CEBPA* axis, circ_0009910, which targets miR-20a-5p and predicts an adverse prognosis, and circBA9.3, a fusion product of *BCR::ABL1*. When circBA9.3 is upregulated, it increases tyrosine kinase activity and consequently resistance to therapy [221, 223, 234, 235].

### Summary of Leukaemia

Leukaemia, the second most frequent blood cancer, can be classified according to lineage (myeloid, lymphoid) and by rate of development (acute, chronic). In order of the number of deaths caused, these are AML, CLL, chronic myelomonocytic, CML, ALL, APL, AMML, HCL, PLL, acute monoblastic/monocytic, CNL and NK leukaemia. Genetically, AML is a highly heterogeneous disease with many variants, the most frequently mutated genes being *NPM1, DNMTA,* and *FLT3*, along with numerous translocations and inversions. Some, such as *RUNX1::RUNX1T,* are linked to a favourable prognosis, while others, such as *DEK::NUP21,* are linked to a poor prognosis. In contrast, almost all cases of APL and CML are linked to a single lesion, t(15;17)(q22;q21) and t(9;22)(q34;q11), respectively, with targeted molecular therapy often highly successful.

As with lymphoma, the majority of ALLs and CLLs arise from malignant B lymphocytes, so immunoglobulin genes are important, often translocated with *MYC* and *BCL2*. In CLL, del(13q14) is present in approximately half of cases (which indicates a better prognosis), mutations in *NOTCH1* are present in a fifth of cases, while the presence of a mutated *BTK* can be treated with a tyrosine kinase inhibitor (TKI). The molecular pathology of ALL is more complex, and B/T cell lineages can be identified, with lesions in *PAX* and *KMT2A*, and the Philadelphia chromosome being common. *BRAF* ^*V600E*^ is the defining lesion in HCL, while mutated *DNMT3, FLT3, IDH1/2, RUNX1,* and *TET2* are common in monocytic leukaemia, and *SRSF2, TET2,* and *ASXL1* lead the frequency of abnormalities in myelomonocytic leukaemia.

There are no clear genetic indicators of NK cell leukaemia; however, CNL is linked to abnormalities in *ASXL1*, *CSF3R*, and *SRSF2*; eosinophilic leukaemia is linked to rearrangements in *PDGFRA, PDGFRB*, or *FGFR1* or *PCM1::JAK2*; basophilic leukaemia is linked to *MYB::GATA1*; and mastocytosis is linked to *KIT* variants. There is considerable evidence for the role of ncRNA variants in the pathogenesis of leukaemia, with some being linked to outcomes; however, none are currently targeted by treatment.

## Myeloma and Related Diseases

The WHO system [25] classifies plasma cell neoplasms and other paraprotein-related diseases into four groups, although the molecular pathology of each group is so far only partially defined:

- Monoclonal gammopathies: cold agglutinin disease, IgM monoclonal gammopathy of undetermined significance (MGUS), non-IgM MGUS, and monoclonal gammopathy of renal significance.
- Diseases with monoclonal immunoglobulin deposition: immunoglobulin-related amyloidosis and monoclonal immunoglobulin deposition disease
- Immunoglobulin heavy chain diseases: the M, G or A classes.
- Plasma cell neoplasms: Plasmacytoma, plasma cell myeloma, and plasma cell neoplasms with an associated paraneoplastic syndrome (i.e., POEMS, TEMPI, and AESOP syndromes).

A key development in our understanding of myeloma was the recognition of a first, and then a second, intermediate developmental stage, i.e., MGUS and smouldering malignant myeloma (SMM), respectively [236, 237]. Accordingly, the ability to determine those patients whose disease will transform to a subsequent stage is highly sought after, an area in which molecular pathology can contribute. For example, should a particular cytogenetic or gene abnormality be present in both an early and subsequent stage, then that abnormality is unlikely to play a role in the transformation. Conversely, should the abnormality be present in a later stage but not in an earlier stage, then that abnormality may be seen as playing a role in the development of the later condition and thus be a marker and/or have pathophysiological significance.

Together, these conditions were linked to 2,774 deaths in England and Wales in 2023, representing 22.7% of all blood cancers [12, 192]. The three conditions in this group are multiple (or sometimes, malignant) myeloma (MM, linked to 2,693 deaths), solitary plasmacytoma (24 deaths) and plasma cell leukaemia (PCL, 57 deaths) [1]. The WHO classification [25] places Waldenstrom’s macroglobulinaemia (WM), which was once considered to be part of the myeloma “family” (and is still considered to be so by some) and which was linked to 101 deaths in England and Wales in 2023, in a separate section with lymphoplasmacytic lymphomas. However, the literature often examines WM alongside MGUS, which, together with SMM, we will explore in the following sections.

### Monoclonal Gammopathy of Undetermined Significance

The key biochemical aspect of all conditions covered in this section is the excessive (<30 g/L) production of an abnormal serum gammaglobulin of a single amino acid sequence arising from a malignant clone of plasma cells. Sometimes described as paraprotein. It can be sub-classified by the particular isotype, i.e., IgM (present in 15% of cases), IgG (70% of cases), IgA (12% of cases), or a biclonal gammopathy (3% of cases). As a result, analysis for a lesion in the gene locus controlling these proteins (*IGH*, at 14q32) is common, although the paraprotein may also be one of the light chains: kappa (coded for by *IGK*, located at 2p11.2) or lambda (*IGL,* located at 22q11.2). A second entry criterion is the presence of <10% clonal bone marrow plasma cells, which can be detected and purified using the CD138/Syndecan-1 marker.

#### MGUS and WM

A link between WM and IgM-MGUS has been noted in that the L256P mutation of *MYD88* (located at 3p22.2 and coding for a transduction adaptor) may be present in approximately 90% of the former, approximately 50% of cases of the latter, 10% of cases of marginal zone lymphoma, and 4% of cases of CLL, but is absent from cases of IgG-MGUS, pointing to its potential use in diagnosis [238]. Other studies have described certain cases of WM as “smouldering,” which is generally taken to mean that they develop slowly, and have reported that the frequency of a panel of abnormalities [+14, del(6q23-25), +12, and +18q11-23] increased progressively from 18% of cases of IgM-MGUS cases to 20% of smouldering WM cases and to 73% of symptomatic WM cases. This suggests a multi-step transformation of clonal B cells that already harbour the phenotypic and molecular features of a malignant WM clone [239]. Similarly, others reported the L256P mutation in *MYD88* to be present in 27%, 80% and in 85% of cases of IgM-MGUS, smouldering WM, and WM, respectively [240].

The observation that 27% of WM patients had a *CXCR4* mutation (located at 2q22.1, which codes for the receptor for the chemokine stromal cell-derived factor 1, also known as CXCL12) was followed by its identification in MGUS, while another study used NGS to identify *KMT2D* mutations (located at 12q13.12, which codes for lysine methyltransferase 2D) in 24% of WM cases and in 5% of IgM-MGUS cases [241–243]. The combination of high frequencies of cells with both *MYD88* and *CXCR4*, compared to low levels of both, gives a hazard ratio (95% CI) of 3.5 (1-4-9.3) for progression to symptomatic WM [244]. The National Genomic Test Directory for England [52] refers to a multi-target NGS panel for detecting small variants in *MYD88* and *CXCR4* in the investigation of WM and MGUS.

#### MGUS and Myeloma

Investigation of the molecular pathology of this condition has been, and continues to be, informed by that of myeloma, which dominates the literature. A key observation in one study was that although the incidence of translocations at 14q32 (the site of the *IgH* locus) was similar, +13 was present in 40% of patients with a myeloma or with PCL, but in only 21% of MGUS cases, suggesting a transformation route from MGUS to myeloma, a finding subsequently confirmed in several studies [236, 245, 246].

Other authors have noted that t(14;20)(q32;q12)/*(IGH::MAFB*) is present in 1.5% of myeloma cases but in 5% of MGUS cases [247], that deletions of *TP53*, although common in myeloma, are absent in MGUS [248], and that chromosome 13 abnormalities are strongly associated with t(4;14)(p16;q32)/(*FGFR::IGH*), which is present in 10.3% of MM cases and in 9.6% of MGUS cases [249]. This has implications for oncogenesis, as 4p16.2 is the location of *FGFR3*, coding for fibroblast growth factor receptor 3, with mutations in this gene having been implicated in several cancers [250]. Multiple cytogenetic abnormalities are common: for example, in one series, t(14q32) and del(13q14) were present in 2% of MGUS cases but in 18% of MM cases [251].

However, not all studies support this: a case-control (243/1285) GWAS analysis of MGUS in a German population reported 10 risk loci on 8 chromosomes, but none of these were significant (p < 0.05) in a parallel study of 294 cases and 272 controls in a Czech population, a finding emphasising the need for caution [252]. The C allele SNP in *ULK4*, at 3p22,1, which codes for a serine/threonine kinase, has an OR (95% CI) of 1.32 (1.02–1.72) for MGUS and 1.39 (1.04–1.86) for myeloma [253]. Sun and colleagues used microarray analysis to probe plasma cell mRNA from 334 MGUS patients, 40 of whom progressed to MM, and found that the downregulation of *IGLV1-44* at 22q11.22, *IGKC* at 2p11.2, *IGHA1* at 14q32.33, *PTPN1* at 20q13.13, and *ECHDC2* at 1p32.3 was linked to progression to MM [254].

### Smouldering Multiple Myeloma (SMM)

This intermediate, proposed in 1980, is defined biochemically by a gammaglobulin level higher than in MGUS (i.e., serum M-protein ≥30 g/L and clonal bone marrow plasma cells *≥*10% and <60%, without other features such as hypercalcaemia). It carries a 10% annual risk of progression to MM [237, 255, 256].

#### Genetics of SMM

One of the first studies reported abnormalities in 251 patients, the most common being a trisomy with an *IgH* translocation in 43.9%, t(11;14)(q13:q32) in 16.2%, and t(4;14)(p16;q32) in 10.3% [255]. The significance of t(11;14) is that it brings together *CCND1* at 11q13.3 with *IgH* at 14q32.22, often leading to high *CCND1* expression. A concurrent review pointed to del13q as a common feature of all stages of the myeloma pathway, with primary genetic events in MGUS being *IGH* translocations, hyperdiploidy, and cyclin D dysregulation, and secondary events in SMM and MM being mutations in *NRAS* (in 24% of cases), *KRAS* (27% of cases) and *BRAF* (4% of cases), with inactivation of *TP53, PTEN*, and *RB1*, and with mutations in the NFκB pathway [256]. Many of these were confirmed, and new data were provided by researchers such as Busteros et al. in a study of 214 patients. These authors reported (in approximate order of frequency) hyperdiploidy (55% of patients), del(13q) (45% of patients), +1q (27% of patients), del(16q) (20% of patients), del(6q), del(14q) (both accounting for ∼13% of patients), del(22q) (12% of patients), del(8p) (10% of patients), del(1p) (8% of patients) del(20p), amp(8q24), and del(17p) (all accounting for ∼5–6% of patients), amp(2p), and del 4q (both accounting for ∼4% of patients), and others. Therefore, this is yet another example of the importance of del(17p), in that the tumour suppressor *TP53* is located at 17p13.1. The most common translocations were t(11;14)(∼12%), t(4;14)(∼10%), and t(14;20)(∼2%), while mutations in *KRAS* (∼13% of patients), *NRAS* (∼6% of patients), and *BRAF, TP53, ATM, DIS3,* and *FAM46C* (all accounting for ∼2% of patients) were the most common gene abnormalities. Biallelic inactivation was present in 6% of patients, primarily involving *TP53, RB1, CDKN2C, ZNF292, DIS3,* and *FAM46C* [257].

#### SMM Progression to MM

Numerous genetic abnormalities have been linked to disease progression, such as those in *MYC, BRAF, FAM46C, NRAS*, t(4;14), t(6;14), and deletions in 1p, 14q, 16q, and 17p, while abnormal cyclins have also been postulated as a common driver. In one study, *KRAS* mutations were associated with a hazard ratio (95% CI) of 3.5 (1.5–8.1) for shorter time to progression. In another study, t(4;14) resulted in a median time to disease progression of 28 months compared to 55 months if t(11;14) was present [255–258]. Despite the importance of single gene/chromosomal abnormalities, there is a desire to combine genes and other factors to develop scoring systems for the risk of disease progression.

Khan et al. presented data from 105 patients on the potential of a four-gene panel to predict SMM progression to MM, these being (in order of predictive power) *RRM2* (located at 2p25.1, coding for ribonucleotide reductase regulatory subunit M2), *DTL* (located at 1q32.3, coding for a ubiquitin protein ligase homolog), *TMEM48/NDC1* (located at 1p32.3, coding for a transmembrane nucleoporin) and *ASPM* (located at 1q313, coding for an abnormal spindle protein homolog) [259]. Using these as a scoring system, a cut-off point was identified for a subset of 14 patients with an 85.7% probability of requiring therapy, compared to the remaining 91 patients whose probability was 17.8%. Botta and colleagues focused on inflammation, suggesting the value of an 8-gene signature (*IL8, IL10, IL17A, CCL3, CCL5, VEGFA, EBI3* and *NOS2*), which could identify MGUS/SMM/MM with 84% accuracy [260]. Other scoring systems, some of which include non-gene features (primarily biochemical), refer to cytogenetics, such as t(4;14), t(14;16), +1q (some 1q21), del 17p (some more precisely 17p13), monosomy 13, and del 13q, in addition to genes with mutations in *TP53, ATM*, *KRAS, NRAS,* and *MYC* [258, 260–262].

### Myeloma

#### Genetics of MM

The earliest reports of cytogenetic abnormalities in MM were of extra bands in 14q, i.e., +14q [263–265], followed by abnormalities at 17p, t(11;14), t(4;14), and +11q13 [249, 266, 267] and *c-MYC, N-RAS, K-RAS,* and *TP53* [268–271]. As mentioned above, t(4;14)(p16.3;q32) provides the basis for at least one transforming event, as it brings together the *IGH* locus and the *FGFR3* proto-oncogene, as does t(11;14)(q13:q32) with *BCL1/CCND1* at 11q13.3, coding for the transcription regulator cyclin D1 (each present in ∼25% of cases), and t(4;14)(p16;q32) with the *WHSC1/MMSET/NSD2* locus at 4p16 [236, 272–274].

The presence (in 45% of cases) of hyperdiploidy, most often of chromosomes 3, 5, 7, 9, 11, 15 and 17, although −13q is considered to be an important pathophysiology event, while del(17p), del(1p) and +1q21 may be secondary events. The majority of the remaining 55% of cases are characterised by reciprocal translocations between *IGH* and a number of oncogenes, such as *FGFR3/MMSET* (t(4;14))*, CCND3* (t(6;14))*, CCND1* (t(11;14))*, MAF* (t(14;16)) and *MAFB* (t(14;20)), [275]. Awada and colleagues summarised the frequencies of *KRAS* and *NRAS* (each present in ∼20% of cases), *FAM46C* and *DIS3* (present in ∼11% of cases each), *TP53* (present in 8% of cases), and *BRAF* (present in 6% of cases), with *TRAF3, LTB,* and *ATM* present in <5% of patients [276]. Several of these were also described by Maura et al., who reported the potential roles of the histone-coders *HIST1H1B/H1-5, HIST1H1D/H1-3, HIST1H1E/H1-4*, and *HIST1H2BK/HSBC12*, all at 6p22, with *FUBP1* at 1p31.1 and *MAX* at 14q23.3, both of which code for MYC-related factors [277].

Several groups have used NGS to recognise candidate genes involved in the development and progression of myeloma. Zhan and colleagues probed CD138^+^ plasma cells from 74 patients with MM, using Affymetrix gene chips to identify 50 downregulated genes (including *SDF1, TNFRSF7, RNASE6, APOC1, DEFA1* and *LYZ*) and 70 upregulated genes (including *CDKN1A, EIF3S9, GMPS, H1F2, LAMC1* and *PTPRK*) [278]. Shaughnessy et al. further demonstrated the strengths of NGS by reporting 70 genes linked to MM, the majority of which were found on chromosome 1 [279]. However, Greenberg and colleagues published a list of 22 genes associated with MM (*BAX, CASP9, CD4, CYP1A1, DNAH11, DNTB, HGF, HPSE, IL-1RN, IL6, IL1A, IL1B, IRS1, ITGA6, KLK3, LAG3, RIPK1, SERPINE1, TRAF3, ULK4, VCAM1,* and *XRCC4*) [280]. Notably, none of these 22 genes are shared with the 70 genes described by Shaughnessy et al., illustrating the difficulty of interpreting certain analyses and, in sum, casting doubt on the particular methods and the comparability of the subjects from whom the samples were obtained.

#### Predicting Disease Progression

The development of overt bone disease has been linked to the over-expression of genes such as *TNFRSF11*A at 18q21.33, which codes for the receptor activator of NFκB (RANK) and its ligand RANKL, coded for by *TNFSF11* at 13q14.11, and to *TNFRSF11B*, at 8q24.12, coding for osteoprotegerin, members of the signalling *NOTCH* and *WNT* families. Meanwhile, mutations in *IL6* and its product, with its receptor, are known to have effects on the bone marrow microenvironment [236] and have a place in the PI3K/AKT/mTOR second messenger pathway [281]. Stein and colleagues compared 182 untreated patients with myeloma, 329 patients undergoing treatment, and 294 patients at or near relapse, finding mutations in *RAS-RAF* in 31.3%, 35.6%, and 46.6%, respectively, *NRAS* in 14.3%, 16.1%, and 24.5%, and *TP53* in 6.6%, 9.7%, and 17% respectively (all p < 0.01). These data point to potential roles for different genes as drivers of disease development, but, notably, there were no trends in *BRAF* ^*V600E*^ *, TRAF3, FGFR3, RB1, CDKN2C, DNMT3A, ATM/ATR, TET2,* or *BIRC3* [282].

A research group from China sequenced 400 plasma cell genes from 50 MM patients, reporting that 76% had a *TP53* mutation, 18% had an *NRAS* mutation, and 14% had a *BRAF* mutation, while low levels of *BCL6* at 3q27.3, coding for a transcription repressor (a mutation also common in B cell lymphomas)*, BIRC3* at 11q22.2, coding for an inhibitor of apoptosis*, HLA-DQA1* at 6p21.32, and *VCAN* at 5q14.2-14.3, coding for a proteoglycan, were linked to a poor prognosis [283]. Uckun and Qazi focused on mRNA for the ERBB isotypes (coding receptor kinases) in 787 patients, reporting no difference in levels of *ERBB1* at 7p11.2, coding for the epidermal growth factor receptor, *ERBB2* at 17q12, coding for HER2, or *ERBB3* at 12q13.2, coding for HER3, according to disease stage. However, patients with the highest tertile of *ERBB2* message had the most adverse outcomes with a hazard ratio (95% CI) of 2.34 (1.30–4.22), although age, serum beta-2-microglobulin, and albumin also affected outcomes [284].

Wallington-Beddoe and Mynott suggested that cytogenetic abnormalities such as trisomies (of odd-numbered chromosomes, present in 40%–50% of patients), t(11;14) (involving *CCND1*, present in 15%) and t(6;14) (involving *CCND3*, present in 5%) all indicate a favourable prognosis. However, they also reported that +1q (the location of *CKS1B,* present in 35%–40% of cases), del(1p) (*FAM46C, CDKN2C* and *FAF1*, present in 30% of cases), and abnormalities in *MYC* at 8q24 (present in 15%–20% of cases) all indicate a poor prognosis, with −13 (affecting *RB1* and present in 45%–50% of patients) indicating an intermediate prognosis, and that the prognosis if t(14;14) is present (affecting *FGFR3* and *MMSET*, present in 15% of patients), is poor to intermediate, although Heider et al. suggested that this translocation indicates a high risk, as do t(14;16) (affecting *MAF,* present in 3%–5% of cases) and t(14;20)(affecting *MAFB*, present in ∼1% of cases) [285, 286]. Black and Glavey considered the presence of t(11;14) and t(6;14) to be a standard risk factor for poor overall survival, whereas the presence of t(4;14), t(14;16), t(14;20), del(17p), +1q, and −13 [275] are considered high-risk factors.

Perhaps unsurprisingly, an increased number of circulating plasma cells indicates a poor prognosis in MGUS and MM [287]. Perroud and colleagues used an NGS panel comprising *CCND1, DIS3, EGR1, FAM46C (TENT5C), FGFR3, PRDM1, TP53,* and *TRAF3*, along with seven hotspots in *BRAF, IDH1, IDH2, IRF4, KRAS,* and *NRAS* in 87 patients with newly-diagnosed MM and 11 patients with relapsed/refractory MM, finding that the mutational load was generally higher in relapsed disease. Despite the very small sample size, *TP53* was the most prevalent mutation, occurring in 13% of patients with newly diagnosed MM but in 81% of patients with relapsed/refractory MM [288]. NICE guidelines NG35 “Myeloma: Diagnosis and Management” suggest using FISH on CD138-selected bone marrow plasma cells for the detection of t(4;14), t(14;16), 1q gain, del(1p) and del(17p)(*TP53* deletion), t(14;20), and the standard-risk abnormalities t(11;14) and hyperdiploidy [289].

The National Genomic Test Directory for England has a section on “Plasma cell dyscrasia” [52]. It describes the use of an NGS multi-target panel for small variant detection in *KRAS, NRAS, BRAF, TP53, DIS3, TENT5C*, and *IRF4*; for structural variant rearrangement detection in *IGH::FGFR3, IGH::CCND3, IGH::CCND1, IGH::MAF, IGH::MAFB*, and *MYC*; and for copy number variation detection with respect to hyperdiploidy, del(1p), +1q, and del(17p). Other analyses, using FISH/RT-PCR, include t(4;14) (*IGH::FGFR3*), t(6;14) (*IGH::CCND3*), t(11;14)(q13;q32) (*IGH::CCND1*), t(14;16) (*IGH::MAF*), t(14;20) (*IGH::MAFB*), and for *IGH* and *MYC* rearrangement, hyperdiploidy copy number, and del(1p), +(1q), and del(17p) (location of *TP53*) copy numbers, all by FISH.

### The Role of Gene Expression Panels in Diagnosis

The astute reader will have rightly observed the markedly complex nature of the preceding section’s molecular pathology, which contrasts with the relative simplicity of CML and *BCR::ABL1*. While numerous commentators have described the transition from MGUS to SMM and MM, there is limited consensus regarding the genes involved in this process [236, 256, 276, 286, 290, 291]. This is illustrated by the figures in Table 17, which show the variability in the proportions of various genetic and chromosomal abnormalities across the dyscrasia spectrum [246, 247, 251, 256, 257, 293].

**TABLE 17:** Genetics of the MGUS/SMM/myeloma pathway.

​	MGUS	SMM	MM
del(1p)	-	2	17
del(8p)	-	7	19
del(13)	25	34	47
del(13q)	25–50	35–50	40–50
del(13q14)	22–24	-	44–55
del(16q)	-	13	17
del(16q23)	6	8	21
del(17p)	-	6	13
del(17p13)	1–22	1	10–42
t(6;14)	0	-	1.1
t (14;20)	3–5	<1	1–1.5
t(4:14)	2–15.5	13	10–18
t(14;16)	2–3	3	2–43
t(11;14)	12	-	17–19
t(14q32) and del (13q14)	3	-	26
+17p13	10	-	14
Hyperdiploidy	15–45	45	45–46
Genomic imbalance*	5/Case	7.5/Case	12/Case
Any *IGH* translocation	40–50	40–50	50–70
t(11;14)(q13;q32)	10–25	10–25	15
t(4;14)(p16;q32)	2–9	3–13	10–15
t(14;16)(q32;q23)	2–5	2–5	2–5
*MYC* rearrangement	3	4–35	15–55
Mutation in *NRAS*	-	4.5	17
Mutation in *FAM46C*	-	0	7
Mutation in *KRAS*	-	13	22
Mutation in MAPK pathway	​	24	44
Mutation in NF*k*B pathway	-	5	16
Mutation in DNA repair pathway	-	7	17
Total number of SNVs per patient sample*	89 (45–115)	-	120 (95–155)

Data are prevalence %, except*. SNV, single nucleotide variation.

From Refs. [246, 247, 251, 256, 257, 290, 292].

Pula and colleagues summarised reports of 10 panels reporting differences in gene expression in MM, eight of which included 15 or more entries [294]. The pooled sample size was 4,431 cases, with panels ranging in size from 4 to 92 genes, whose expression predicted clinical outcome. Of the 385 genes listed, 29 (Table 18) were present in two or more panels: *BIRC5* was present in five panels, and *LTBP1* was present in three panels. Notably, the majority (n = 12, 41%) of genes generate products with roles in mitosis. This summary points to the potential value of a further panel to help identify those patients at risk of a poor outcome.

**TABLE 18:** Genes linked to MM outcomes.

Gene	Location	Product
*AHCYL1*	1p13.3	Adenosylhomocysteine
*AIM2*	1q23.1	An interferon-inducible protein with a role in apoptosis
*ALDOA*	16p11.2	Aldolase
*ASPM*	1q31.3	Abnormal spindle-like microcephaly-associated protein; required for mitotic spindle formation
*BIRC5*	17q25.3	Survivin: an inhibitor of apoptosis
*BUB1B*	15q15.1	A serine-threonine kinase with a role in spindle assembly in mitosis
*CDC2*	10q21.2	Cyclin-dependent kinase 1, involved in cell cycle regulation
*CKS1B*	1q21.3	A subunit of a regulatory protein of cyclin-dependent kinases
*DLG7* ^*a*^	14q22.3	A protein required for microtubule function and gene stability
*ESPL1*	12q13.13	Separase, a cysteine protease, required for the segregation of sister chromatids
*FAM49A*	2p24.2	Unclear: but has a role in orofacial clefts
*KIAA1754*	10q25.1	A protein that interacts with the inositol 1,4,5-trisphosphate receptor
*KIF14*	1q32.1	A member of the kinesin family, molecules that interact with microtubules
*KIF20A*	5q31.2	A member of the kinesin family, molecules that interact with microtubules
*LARS2*	3p21.31	An aminoacyl-tRNA synthetase
*LTBP1*	2p22.3	A protein that binds to TGF-β
*MAGEA6*	Xq28	An activator of a ubiquitin ligase linked to the repression of autophagy
*MCLC* ^*b*^	1p13.3	A chloride channel
*MCM6* ^*c*^	2q21.3	A regulator of DNA replication
*MPHOSPH1*	10q23.31	A member of the kinesin family, molecules that interact with microtubules
*NCAPG*	4p15.31	A subunit of condensin, a molecule involved in chromosome condensation
*PSMB4*	1q21.3	A subunit of the 20S proteasome
*PSMD4*	1q21.3	A subunit of the 20S proteasome
*RACGAP1*	12q13.12	An activator of a GTPase
*TBRG4*	7p13	A regulator of TGF-β
*TMPO*	12q23.1	Thymopoietin
*TOP2A*	17q21.2	DNA topoisomerase II-α
*YWHAZ*	8q22.3	A regulator of apoptosis
*ZWINT*	10q21.2	Involved in kinetochore function, linking microtubules in mitosis

From reference [294]. ^a^Also known as DLGAP5. ^b^Also known as CLCC1. ^c^Also known as KIF20B.

### Plasma Cell Leukaemia (PCL)

This final stage of the plasma cell dyscrasia lineage was described in detail in the 1940s. One of the earliest identified cytogenetic abnormalities is in chromosome 14 [264, 265, 295, 296], and at the gene level, in *MYC, FGFR,* and *Ras* [271, 297–300], as described above in MGUS, SMM and MM. However, statistically relevant data are, in many cases, marred by the small numbers of cases, a consequence of the rarity of this disease. Abnormal cytogenetics were reported in 24% of cases of MGUS, 33% of MM cases, and 50% of PCL cases, suggesting a likely pathophysiological significance trend [301]. Ana and colleagues found that, in a comparison of MGUS, MM, and PCL, the frequency of deletions in chromosome 13 increased sequentially at 21%, 38%, and 75%, respectively, pointing to the possibility of a transformation driver in this chromosome [302].

Similarly, Chang et al. reported the frequency of amplification of 3–8 copies of *CKS1B* at 1q21 (coding for a cyclin-dependent kinase subunit) to be 0% in MGUS, 36% in MM at diagnosis, 52% in MM at relapse, and 62% in PCL [303], a further trend of possible significance and noted in the WHO guidelines [25]. The authors subsequently compared PCL and MM, reporting an increased del(17p) (37% v 11%, p = 0.001), del(13q) (63% v 41%, p = 0.02), del(1p21) (33% v 18%, p = 0.03), amp-1q21 (51% v 34%, p = 0.05), t(4;14) (29% v 13%, p = 0.05), and t(11;14) (27% v 13%, p = 0.11) [304]. De Larrea and colleagues summarised the median frequencies of cytogenetic abnormalities in PCL as follows: hypodiploidy in 42%, hyperdiploidy in 7%, a complex karyotype in 59%, del(13q14) or −13 in 63%, del(17p13) in 18%, t(11;14) in 37%, t(4:14) in 4%, and t(14;16) in 8% [305]. Chang et al. noted that patients with PCL with del(1p21) or t(4;14) had shorter overall survival: 6.2 months compared to 33.5 months for those without the abnormality (p = 0.006) with the deletion and 1.5 months compared to 21.6 months (p = 0.003) with the translocation [304].

#### Primary and Secondary PCL

PCL may be classified as primary (pPCL), where it arises without prior evidence of a MM, or secondary (sPCL), where it is known to arise from pre-existing MM. Mosca et al. used FISH on pathological samples from 23 patients with pPCL, finding that87% of cases harboured an *IGH* translocation, with the most common being t(11;14) in 40% of cases and t(14;16) in 30.5% of cases, with abnormalities observed in 1p (38%), 1q (48%), 6q (29%), 8p (42%), 13q(74%), 14q (71%), 16q (53%), and 17p (35%) [306]. They also reported a biallelic deletion in 8p21.2, the location of *PPP2R2A*, coding for a protein phosphatase subunit, which the authors considered to belong to a family of putative tumour suppressors. Gowin and colleagues summarised the cytogenetics as pPCL being commonly hypodiploid and the *IgH* translocation t(11;14) the most prevalent, with sPCL more likely to be hyperdiploid (having possibly evolved from an MM clone) and with more diverse *IgH* translocations, such as t(11;14), t(4:14) and t(14;16), and so on [307].

Tiedemann and colleagues compared 39 cases of sPCL with 49 of pPCL and 439 cases of MM, finding the frequency of hypodiploidy to be 42%, 60% and 40%, respectively, and hyperdiploidy to be 17%, 0% and 60%, respectively. Despite the small number of cases, the difference in the presence of t(11;14) in 71% in pPCL cases and in 23% in sPCL cases was statistically significant [308]. The cytogenetics of pPCL and MM have been reported by three research groups, the most consistent results being the increased frequencies of t(11;14), t(14;16), and del(17p), the latter two showing the greatest increases (Table 19). This supports the hypothesis of a transformation driver at one or both of these sites [309–311]. Gundesen et al. [309] and Lionetti et al. [311] also reported amp-1q/1q gain in 32%/48% of pPCL cases and 40%/51% of MM cases, respectively. The latter research group also reported t(14;20) frequencies of 5% and 3%, respectively. Avet-Loiseau et al. [310] also reported that t(11;14) and del(17p) were linked to a poor prognosis in pPCL, while Tiedeman found that *MYC* translocations were linked to poorer survival in pPCL [308]. Chang reported outcomes in 14 patients with pPCL (with an overall survival of 45 months) and 26 with sPCL (with an overall survival of 19 months), which may be a false negative (p = 0.09) due to the small sample size [304].

**TABLE 19:** Genetics of pPCL and MM.

	Gundesen et al. [309]		Avet-loiseau et al. [310]		Lionetti et al. [311]	
	pPCL	MM	pPCL	MM	pPCL	MM
t(11; 14)	26%	21%	25%	20%	39%	23%
t(4:14)	14%	14%	21%	15%	13%	18%
t(14; 16)	20%	4%	17%	3%	30%	5%
del-17p	40%	11%	20%	7%	35%	5%
del-13q	42%	48%	65%	45%	74%	56%

Todoerti and colleagues used the power of Affymetrix NGS to probe total RNA from 21 pPCL cases and 55 MM cases, identifying a 503-gene panel that could distinguish between the two types of cancer, and a further 27-gene panel with potential clinical relevance. Of these 27, 10 (*PECAM1* (at 17q23.3), *MKX* (10p12.1), *CALCRL* (2q32.1), *C3orf14* (3p14.2), *ALDH1L2* (12q23.3), *WARS* (14q32.2), *SLC15A2* (3q13.33), *RNU5D* (1p34.1), *CTH* (1p31.1), and (1p32.1)) were linked positively to survival, with 17 (*FAM111B* (at 11q12.1), *MCTP1* (5q15), *C10orf10* (10q11.21), *FNBP1* (9q34.11), *EFEMP1* (2p16.1), *FAIM3* (1q32.1), *CPEB4* (5q35.2), *EDN1* (6p24.1), *PVALB* (22q12.3), *LY86* (6p25.1), *LAPTM5* (1p35.2), *PARP15* (3q21.1), *PLEKHF2* (8q22.1), *PDK4* (7q21.3), *TNFAIP3* (6q23.3), *FAM105A* (5p15.2), and *TCN2* (22q12.2) linked negatively to survival [312].

A further study analysed the expression levels of genes across the spectrum of plasma cell dyscrasia, including normal plasma cells, MGUS, SMM, MM, and PCL. A clear downward sequential trend was found across the five groups for *BTN3A2, C13oft15, CD36, FOS, TSTL1,* and *MGC29506*, and a clear upward expression trend was found for *BUD31, C16orf42*, *PSMD6, CDC42SE1, RHOA, CORO1A, EIF1B, GNB2, MRPS18A, MVP,* and *MYL12B.* *EZH2* expression was linear between normal controls, MGUS and SMM, then increased in a stepwise manner for MM and PCL, while *ILK* expression increased only in PCL. Compared to expression in normal controls, *PRG3* expression was lower in MGUS, SMM and MM, but reduced in PCL. These data provide fascinating insights into the role of certain genes in the natural history of plasma cell dyscrasias, from health to frank leukaemia. The National Genomic Test Directory for England has a section on plasma cell dyscrasias, but does not refer to PCL or either of its subtypes [52].

### Non-Coding RNAs (ncRNAs) in Myeloma and Related Conditions

#### miRNAs

One of the earliest and most thorough investigations of these molecules, by Pichiorri and colleagues, used mRNA and miRNA microarray chips and RT-PCR to probe 49 MM-derived cell lines and samples from 6 normal donors, 6 patients with MGUS and 16 patients with MM [313]. The principal findings were 5.8-fold–15.6-fold increases in the expression of miR-21, miR181a, miR-93, miR-106b, miR-25, and miR-106a, and a 0.15-fold decrease in the expression of miR-328 in MGUS. Similarly, in MM, there were 4.6-fold–288.9-fold increases (depending on whether they originated from plasma cells or from transformed cell lines) in the expression of miR-25, miR-32, miR-20a, miR-93, miR-106b, miR-106a, miR-181a, miR-21, miR-19a, miR-19b, miR-181b, and miR-92a, with a 0.48-fold reduction in the expression of miR-328. The authors speculate that these abnormalities may contribute to malignant transformation by factors such as promoting plasma cell survival and blocking apoptosis. Some miRNAs, such as miR-21 and miR-32, are differently expressed in MGUS and MM, with others, such as miR-221 and miR-222, exhibiting similar levels in both conditions [313, 314]. Chi et al. built on these cross-sectional studies, probing samples from 33 MM cases, 5 MGUS cases and 9 controls, reporting that 109 miRNAs were upregulated and 20 were downregulated (on average twofold) in MM relative to controls [315].

Jones and colleagues described a microarray/TaqMan RT-PCR method for detecting serum miRNAs, using it to suggest that miR-720, miR-1308 and miR-1246 have potential as diagnostic biomarkers in MM and that combinations such as miR-720 and miR-1308, and miR-1246 and miR-1308 can distinguish between different groups of cases and controls [292]. This theme was extended by Kubiczkova et al., who used TaqMan/qPCR methods to report altered miRNAs, such as miR-34a and let-7e, in MGUS and MM, but also to show that levels of miR-744 and let-7e are linked to overall survival and time to progression [316] (Table 20). Similarly, Li et al. concluded that serum miR-134-5p, miR-107, and miR-15a-5p are also potential biomarkers in MGUS and MM [317]. Lionetti et al. [311] reported 423 upregulated and 41 downregulated miRNAs in pPCL compared to MM. Of these, relative (high/low) expression of miR-22, miR-146a, miR-92a, and miR-330-3p was found to be linked to progression-free survival (PFS).

**TABLE 20:** miRNAs and survival.

	miR-744	let-7e
One-year mortality rate^a^	“Low” expression: 41.9% “High” expression: 3.3%	“Low” expression: 34.6% “High” expression: 3.9%
Median time to remission^a^	“Low” expression: 11.5 months “High” expression: 47.5 months	“Low” expression: 11.5 months “High” expression: 47.5 months
Cox proportional hazards survival model	0.67 (0.55–0.82)^b^ P < 0.0001	0.61 (0.45–0.83)^b^ P = 0.002
Cox model for prognostic impact in time to progression	0.69 (0.58–0.82) P < 0.0001	0.55 (0.43–0.72) P < 0.0001

^a^ All high/low differences are significant at p ≤ 0.001. High/low expression defined by the area under the receiver operating characteristic curve.

^b^ Hazard ratio (95% confidence interval). From reference [292].

#### lncRNAs

The power of NGS was once again demonstrated by Ronchetti and colleagues, who used an Affymetrix GeneChip array and qRT-PCR to probe CD138^+^ plasma cells from 268 individuals representing the spectrum of plasma cell dyscrasias [318]. The expression of 21 lncRNAs was progressively deregulated across the spectrum. Examples include RLIM-6 at Xq13, with a correlation coefficient of 0.37; ANGPTL-1 at 1q25, with a correlation coefficient of 0.3; and SERPINC1-1, also at 1q23, with a correlation coefficient of 0.29, suggesting roles in disease progression. A regulatory role may be present as these three lncRNAs are antisense to *SCL16A2, RALGPS2*, and *ZBTB37,* respectively. Butova et al. used a similar approach, with Illumina NGS and RT-qPCR to report that 52 different lncRNAs were significantly deregulated between MM and PCL samples [319]. Of these, the expression of both LY86-AS1 and VIM-AS1 was significantly reduced in PCL compared to MM, suggesting possible links with disease progression.

NGS and qRT-PCR were also used by Todoerti and colleagues to focus on lncRNAs in MM and pPCL patients with t(11;14). These authors found 38 lncRNAs to be differentially expressed, with three increased and 35 decreased in pPCL [320]. Furthermore, lower expression of Linc00886 was linked to adverse PFS and overall survival, NINJ2-AS1 and Linc02728 to poor PFS, while higher expression of SNHG6 was linked to poor overall survival, although in multivariate analysis, only the latter remained a significant predictor of overall survival. Data from Li and colleagues reported increased expression of the lncRNA UCA1 in MM, which correlated inversely with that of miR-331-3p. Other data pointed to a role of UCA1 in suppressing apoptosis, thus promoting proliferation [321].

Yang et al. reviewed lncRNAs in MM, showing their relationships with miRNAs, their effect on genes such as *MYC* and on second messengers, and their roles in PFS and overall survival, several of which, such as ANRIL and HOTAIR, have roles in numerous malignancies [322]. A review by Lei and colleagues summarised the potential of lncRNAs such as PCAT1 and LINC0017 as therapeutic targets and described the mechanisms by which lncRNAs and miRNA may act on genes to effect malignant transformation [323].

#### Other ncRNAs

Although only recently (in terms of miRNAs and lncRNAs) described in depth, numerous roles for circular RNAs (circRNAs) have been reported. One of the earliest circRNAs to be discovered, circ_0000190, was found to be downregulated in MM tissues and in plasma compared to controls, and high/low expression levels were linked to PFS and overall survival. A possible mechanism for this may be via repression of miR-767-5p and the MAPK4 transduction pathway [324]. Similarly, Liu et al. reported increased expression of circRNA-101237 in MM, especially in those with cytogenetic abnormalities del(13q14), amp(1q21), del-p53, t(4;14) and t(11;14), and that high levels were linked to poor PFS and overall survival [325]. Other studies have shown the possible influence of circRNAs on drug resistance, on bone metabolism, and as tumour suppressors [326–328]. Mirazimi et al. reviewed circRNAs in MM, Peres and colleagues reviewed circRNAs in leukaemia, lymphoma and MM, and Lei et al. summarised data on small interfering RNAs in MM, such as IONIS-AR-2.5Rx, which may target *GALNT2* at 1q42.13, a gene that codes for a metabolic enzyme [235, 323, 329].

### Summary of Myeloma and Related Diseases

Although they are the least common blood cancer, plasma cell dyscrasias have the worst prognosis, with a global survival index (mortality/incidence) of 0.65 in MM, compared to leukaemia (0.63) and lymphoma (0.43); the comparable data in the United Kingdom are 0.56, 0.5, and 0.33, respectively [1, 25]. While the MGUS-SMM-MM-PCL progression, proposed over 25 years ago [236, 301], is now acknowledged, the majority of cases are diagnosed at the MM stage, when the disease becomes symptomatic, with consequent difficulty in confirming disease progression [286, 330, 331]. However, the fortuitous discovery of generally asymptomatic MGUS and SMM has provided the opportunity to include molecular genetics in the list of other laboratory investigations, most notably of paraproteins and other molecules such as beta-2-microglobulin.

A key feature of these conditions, as evidenced by the length of this text, is the complexity of the biology of the malignancy, and so lack of clarity, as shown in Tables 17 and 18, and elsewhere, such as there being no genes in common in the panels of Shaughnessy et al (70 genes, 280) and Greenberg et al (22 genes, 281). Almost all laboratory analyses have considerable overlap between the stages, although there are some examples of differential expression of genes that may bring useful light on our understanding of these diseases.

## Myeloproliferative Neoplasms and Other Blood Cancers

We return to malignancies of the GEMM stem cell and the WHO classification [91], which provides an umbrella descriptor of myeloproliferative neoplasms (MPNs). Having already covered granulocytic and monocytic leukaemias, the main remaining conditions are polycythaemia vera (PV), essential thrombocythaemia (ET), and primary myelofibrosis (PMF). Somewhat confusingly, the classification introduces the term “myelodysplastic neoplasms” to replace “myelodysplastic syndromes”, but the abbreviation “MDS” is retained. The classification also refers to myeloproliferative neoplasms not otherwise specified, which can be considered a category for those rare conditions that do not easily fit into one of the four major groups. This section will also consider cancers of the red cell lineage, histiocytic/dendritic cell neoplasms and paediatric blood cancers.

### Polycythaemia Vera (PV)

Although not evident from its descriptor, PV is taken to primarily imply an increase in red cell mass but may develop into a true “poly” condition with an increase in other cell lineages. A further issue is the implied malignant aspect of PV, in contrast to erythrocytosis, where a high red blood cell count is generally the result of an external factor, such as hypoxia or increased levels of erythropoietin (EPO). First described in 1892, clinical and laboratory reports of PV can be traced to the early 20th century; however, it was not until the 1950s that fully detailed reports emerged, some of which were linked to leukaemia [332–335].

Perhaps the earliest report of abnormal cytogenetics in PV is that of Modan and colleagues, who reported 8% of analysed cells to be hypodiploid (compared to 2.5% in controls) and 3.3% to be hyperdiploid (compared to 0.7% in controls), while Zech et al. reported a series of 10 patients, four of whom had trisomy 9, one had trisomy 8, and two had del(20q), which was later mapped to 20q12 [336, 337]. Subsequent studies focused on mutations in the EPO receptor and its downstream signal transduction pathway involving tyrosine kinase JAK2 (coded for by *JAK* at 9p24.1) and members of the STAT family, which are also known to be linked to AML [338–341]. Additional analyses reported *PRV-1*, subsequently found to be CD177, expressed by neutrophils and encoded by *CD177* at 19q13.31, with marked mRNA overexpression of PRV compared to secondary erythrocytosis, and c-Mpl (CD110), the receptor for thrombopoietin, coded for by *MPL* at 1p34.2 [342–345]. NGS methods identified mutations in *TET2*, *DNMT3A* and *ASXL1*, but not in *CALR* at 19p13.13, coding for the calcium-binding lectin chaperone (and transcription regulator) calreticulin [346, 347]. However, the leading genetic lesion in PV is the exon 14 lesion *JAK2* ^*V617F*^ (valine to phenylalanine); a mutation in exon 12 is less frequent. The allele burden of the former is linked to clinical and laboratory features–spleen size (p < 0.001), red blood cell count (p = 0.004) and the white blood cell count (p = 0.001), but not platelet count (p = 0.860), in addition to a risk of myelofibrotic progression (p < 0.001) and transformation to leukaemia (p = 0.03) [348–350].

#### Chuvash Polycythaemia

Described in detail in 1997 [351], this fascinating condition provided the opportunity to directly link a precise genetic lesion with clinical and laboratory features. *VHL* at 3p25.3 codes for the 24 kDa Von Hippel Lindau protein that binds to the hypoxia-inducible factor 1α (HIF-1α), coded for by *HIFA* at 14q23.3, which itself has many roles, including as an important regulator of the body’s response to hypoxia, and therefore, the levels of EPO [352]. Loss-of-function mutations in *VHL* lead to the over-expression of HIF-1α, which is the basis of polycythaemia, and acts as a tumour suppressor [353, 354]. The most common form of polycythaemia (C598T) is estimated to have arisen from a single founding event 14,000 to 62,000 years ago, while *VHL* ^*R200W*^ may provide heterozygous protection against anaemia, thus explaining its persistence [355–357].

### Essential Thrombocythaemia (ET)

Characterised by a very high platelet count (e.g., >600 × 10^9^/L), ET was reviewed by Smith over 70 years ago, recognising the clonal nature of the disease. Cytogenetic abnormalities (such as trisomy 1q and the Philadelphia chromosome) were described in the 1980s [358–361]. Subsequent reports described del11q21 and del5q, with the potential involvement of thrombopoietin (coded for by *THPO* at 3q27.1) and its receptor c-Mpl [362–364]. The developing literature refined these data, with the diagnostic criteria calling for features such as a platelet count >600 and an absence of the Philadelphia chromosome or evidence of the myelodysplastic syndrome (del(5q), t(3;3)(q21;q26) or inv(3)(q21q26)). Meanwhile, Harrison emphasised the complexity of the genetic aetiology of ET with respect to PV and other myeloid neoplasms, regarding *MPL* as a candidate oncogene and considering the excessive platelet count to be the consequence of increased megakaryocytopoiesis [365–367].

In 2005, several research groups reported the presence of *JAK2* mutations in ET (as in PV and other myeloproliferative disorders) [368–371]. While levels of haemoglobin and haematocrit were higher and the risk of thrombosis greater, the platelet count was lower in patients with *JAK2* ^*V617F*^ compared to those free of this mutation [372, 373]. Other mutated genes in ET are shown in Table 21, while *SH2B3, ZRSR2, CSF3R, EZH2, TP53, SRSF2, SETBP1, RUNX1, KIT, U2AF1, CBL, FLT3, IDH2,* and *NRAS* are present at a frequency of ≤3%. Notably, many of these genes are also mutated in PV and myelofibrosis [374–377]. The 2016 classification of myeloid neoplasms includes the presence of *JAK2, CALR,* or *MPL* mutations in its criteria for ET, although the 2022 version does not specifically refer to the genetics of ET [91, 159]. Other scholars have pointed to *JAK2* ^*V617F*^, mutations in *MPL,* and an exon 9 *CALR* indel as signalling drivers in ET [375–377]. However, the sum of these three markers fails to reach 100%, leading to “triple-negative MPN,” which, in the case of ET, accounts for 14%–32% of cases, leaving considerable room for other markers to be defined [375, 377].

**TABLE 21:** Mutated genes in ET.

Gene	Location	Product	Frequency
*CALR*	19p13.13	Calreticulin	∼25%
*TET2*	4q24	A methylcytosine dioxygenase	10%–15%
*ASXL1*	20q11.21	A transcription regulator	5%–10%
*DNMT3A*	2p23.2	DNA methyltransferase 3-α	5%
*SF3B1*	2q33.1	A subunit of an RNA splicing complex	3%
*CEBPA*	19q13.11	A transcription factor	3%–4%
*MPL*	1p34.2	The thrombopoietin receptor	3%–4%

The power of an NGS panel was demonstrated by Sobieralski and colleagues, who probed peripheral blood genomic DNA samples for 37 genes (almost all of those described above) in 36 ET cases and 13 PV cases [378]. Although they were unable to report by diagnosis, the most frequent mutations overall were in *JAK2* (67% of patients), *CBL* (41% of patients), *RUNX1* (32% of patients), *CALR* (26% of patients), and *DDX41* (26% of patients), with only 22% of patients carrying only one mutation, 43% carrying two, 27% carrying three, and 8% of patients carrying four or more mutations. The authors obtained a second sample after a mean of 109 months, finding that half of the patients retained their mutations, the remainder losing two or more, and while no new mutations were found in 32% of patients, a new mutation appeared in 38% of subjects, two new mutations appeared in 24% of patients, and 6% of patients developed three or more new mutations. These important data underline the complexity of the genetic landscape of these two diseases, with fluctuations in the presence of mutations, and so demonstrate the dynamic process of disease progression.

#### Megakaryoblastic Leukaemia

Having discussed excessive megakaryocytopoiesis as the root lesion in ET, a discussion of megakaryoblastic leukaemia naturally follows. First described in the mid-20th century, a genetic abnormality was reported 50 years ago, with strong links to myelofibrosis subsequently published, and an early report of three cases pointing to 11%–75% megakaryoblasts and platelet counts of 35–53 × 10^9^/L [379–383]. Breton-Gorius used monoclonal antibodies and electron microscopy to note the close association between megakaryoblastic leukaemia and acute erythroblastic leukaemia, while Cuneo and colleagues reported numerous chromosomal abnormalities, such as −5/5q, −7/7q, +8 and +21, rearrangements in 3q21 and 3q26, inv(16)(pl3;q22), t(13;20)(ql3 or l4;ql1), and der(7)t(7;17)(p14;q22) [384, 385].

In an elegant series of experiments, Terui and colleagues provided details of the precise cell biology and aetiology of the disease. They found that a soluble product of megakaryoblasts stimulates collagen production by bone marrow fibroblasts and that this soluble factor is transforming growth factor-β. The authors also found that the megakaryoblasts express substantially increased levels of their mRNA [386]. Other authors have reported potential roles for additional growth factors, PDGF and FGF, although these may have a role in the myelofibrosis aspect of the disease [387]. Genes mutated in megakaryoblastic leukaemia include *GATA1* (especially in G21 trisomy associated with Down’s syndrome, where *RUNX1* is also implicated), a fusion of *RBM6* to *CSF1R, KIT,* and *FLT3, JAK2, JAK3,* and *MPL* [388–392].

### Primary Myelofibrosis (pMF)

In this condition we move from leukocytes to the infrastructure of the bone marrow. An expression coined in the Victorian era, numerous case reports of MF demonstrated fibrotic deposits in the bone marrow, with perhaps the earliest cytogenetics report appearing in 1970 [393–397]. These were followed by more complex analyses, such as a report of +8, t(1;6)(q23;21), and t(1;4)(p32;35) [398–400]. A later report of 106 cases found an abnormal karyotype in 35% of cases, with the leading abnormalities being del(13q), del(20q), partial +1q, +8, and del(7q) [401]. As discussed above, the role of growth factors in MF and other myeloid neoplasms is now recognised, and they are likely to drive the malignancy, forming a model that explains the basis of the disease [386, 387, 402–404]. Given the complex nature of the molecular genetics of the myeloid neoplasms, it is not surprising that *JAK2* ^*V617F*^ is also present in many cases of pMF, as are mutations in *MPL, TET2, ASXL1, CBL, ISH1, RUNX1, RB1, TP53, DNMT3A, SRSF2, NFE2, CALR, U2AF1,* and *SF3B1* [405]. The fact that all these genes have already been described in other blood cancers underlines the difficulty of determining their relevance to a particular clinical phenotype.

A decade ago, chromosomal abnormalities such as a complex karyotype or one or more of  + 8, −7/7q, i(17q), −5/5q, −12p, inv(3), or 11q23 rearrangement were beginning to be incorporated into scoring systems for the risk of disease progression and poor survival outcomes [406]. Studies have shown that mutations in certain genes, such as *ASXL1, EZH2, SRSF2,* and *IDH1/IDH2*, bring an increased risk of transformation to AML and reduce survival rates [407, 408]. These five genes were subsequently incorporated into a risk-stratification scoring system [409]. Barosi and colleagues followed up 244 pMF-fibrotic-type patients for a median of 42 months, finding that heterozygosity for *JAK2* ^*V617F*^ was protective against blast transformation compared to homozygosity or the wild-type for this abnormality [410]. Using a similar design, Tefferi et al. probed 254 patients for mutations in *JAK2, CALR, MPL*, and other genes and monitored them for up to 16 years. Overall survival was poorest in those triple negative for a mutation (median survival 2.5 years), intermediate in those with a *JAK2* or *MPL* mutation (4.3 years and 4.1 years, respectively), and best in those with a *CALR* mutation (8.2 years). An additional study found a mutated *ASXL1* to be unfavourable [411].

Although abnormal *JAK2, CALR,* and *MPL* mutations are considered driver mutations, triple-negative disease (present in approximately 9% of cases) leaves room for additional factors (some of which are described above), with multiple mutations often present – 50% of cases have two, 19% have three, 12% have four, and 18% have five or more. At the chromosomal level, −7, inv(3), i(17q), +21, +19, del(12p), and −11q are linked to a high-risk prognosis, whereas a normal karyotype and +9, del(13q), del(20q), and 1q abnormalities are favourable [412–414].

### Myelodysplastic Neoplasms (MDS)

As clinical science advances, the attempts to classify increasingly rare conditions become more complex, and revisions over time are therefore expected to be common, as exemplified by the MPNs. The aetiology developed from the 1980s onwards, with reports of micromegakaryocytes, suggestions of a role for oncogenes, persistent cytopenia, and chromosomal abnormalities [415–419]. While reports of the latter increased [420, 421], one of the first reports of genetic abnormalities was of *AML1/RUNX1*, followed (inevitably) by *JAK2* ^*V617F*^, and by *SF3B1, CEBPA, TERC, TERT, GATA2,* and others [422–426]. Unlike the other MPNs, which consider only three major drivers, Cazzola et al. concluded that there are many driver mutations in MDS, including those in *TET2* (with an incidence of 20%–25%)*, SF3B1* (18%–30%)*, ASXL1* (15%–25%), *DNMT3A* (12%–18%)*, SRSF2, RUNX1* (both 10%–15%)*, U2AF1* (8%–12%)*, TP53* (8%–12%)*, ZRSR2* (5%–10%), with *IDH1/2, STAG2, BCOR, EZH2, JAK2, CBL, NRAS*, *KRAS*, and others (all <5%) [427, 428]. Many of these genes are referenced in the 2022 WHO classification [91]. The number of mutated genes is a strong indicator of overall survival, with the 2-year survival in those free of a *TP53* mutation being approximately 40%, compared to <5% in patients with a mutation [428].

As with other conditions in this section, MDS may progress to AML, and in this respect, longitudinal follow-up data showed an inferior survival outcome (which included AML transformation) for a mutated *BCOR*, with a hazard ratio (95% CI) of 3.3 (1.4–8.1) [426]. Chen-Liang summarised somatic mutations linked to outcome, reporting that *TP53, RUNX1,* a frameshift in *BCOR, SRSF2, USAF1, IDH2, EZH2, ASXL1, STAG2,* and *SETBP1* are linked to decreased survival outcomes, with several also being linked to a shorter time to AML [429]. The scoring system by Schanz and colleagues [430], based on 2,902 patients, placed patients into one of five groups according to their survival outcome:

- Very good outcome: del(11q) or -Y, with a median survival of 60.8 months
- Good outcome: a normal karyotype, del(5q), del(12p), and del(20q) (all as a single anomaly) and double abnormalities including del(5q) (median survival of 48.6 months)
- Intermediate outcome: del(7q), +8, i(17)(q10), +19, +21, any other single abnormality, independent clones, double abnormalities not harbouring del(5q) or −7/7q (26 months)
- Poor outcome: inv(3)/t(3q)/del(3q), −7, double abnormalities including −7/7q, and complex (i.e., three abnormalities) (median survival of 15.8 months)
- Very poor outcome: complex abnormalities (i.e., four or more abnormalities) (median survival of 5.9 months).

The pathology of MDS and its relationship with AML amply demonstrate the complexity of the MPNs [431]. Veiga and colleagues [432] argued for a further layer of intricacy, suggesting that in some cases MDS may arise from clonal haematopoiesis of indeterminate potential (CHiP), or from clonal cytopenias of undetermined significance (CCUS). The latter is associated with potential progression to AML and MDS [91, 433–436]. The 2022 WHO MDS classification subtypes focus on bone marrow and peripheral blood blasts, -5q, −7/7q, *SF3B1* and *TP53* [91].

### Acute Erythroid Leukaemia (AEL)

The 2022 WHO classification places this cancer in the category “AML, defined by differentiation” (i.e., an excess (≥80%) of proerythroblasts, erythroblasts and normoblasts/nucleated red blood cells). It can arise *de novo* or in a secondary manner from pMF, MDS, or ET [91, 437, 438]. Leading cytogenetic abnormalities include monosomy of chromosomes 5 and 7, del(5q) and del(7q), der(1;7)(q10;p10), and t(8;16)(p11.2;p13.3), while an unfavourable outcome may be linked to complex karyotypes and abnormalities in 11q and 17p (with *TP53* being located at 17p13.1), +8, +13, inv(3q), and deletions in all or part of the q arm of chromosomes 5, 7 and 20 [439, 440].

The primary genetic abnormality to be recognised in AEL is in *TP53* (present in 97% of cases, with monoallelic in 68% and biallelic in 29%), with rare cases involving *FLT3* and *NPM1*, and (again, almost inevitably) in *JAK* (present in 12% of cases), but also in *NRAS* (in 19% of cases)*, DNMT3A* (in 9% of cases)*, TET2* (three variants, with frequencies of 43%, 41%, and 37%)*, SF3B1* (in 15% of cases) and *CSFR3* (in 5% of cases) [439, 441, 442]. Fagnan et al. reported on the importance of the erythropoietin receptor (i.e., *EPOR*, at 19p13.2), the activation of which leads to downstream signalling to the STAT-PI3K-MAP pathway, and so the potential for erythroleukaemia [443], while Takeda and colleagues reported gains and amplifications in *EPOR* and *JAK* in a subgroup of patients [444]. The National Genomic Test Directory for England does not refer to erythroid leukaemia.

### Histiocytosis

A leading downstream manifestation of monocytes/macrophages is the histiocyte, a malignant form of which can form tumours in a number of tissues and organs [445–447]. The 2022 WHO classification of histiocytic/dendritic cells recognises three major variants, which are discussed below [91].

#### Plasmacytoid Dendritic Cell Neoplasms

These conditions were originally thought of as being a form of lymphoma, often presenting with dermal manifestations and defined by immunophenotyping [448–452]. Cytogenetics has since pointed to abnormalities in 5q, 6q, 9 (such as del 9p21.3, which is the location of tumour suppressors *CDKN2A* and *CDKN2B*), 12p, 13q, and 15q, with +7q, +22, and del(3p). Molecular genetics may indicate lesions in *TET2*, *TP53, NPM1, FLT3, IKZF1, SRSF2,* and the proto-oncogene *MYC*, the latter often forming a t(6;8)(p21;q24) [451–455].

#### Langerhans Cells and Other Dendritic Cell Neoplasms

A presumptive diagnosis of Langerhans cell histiocytosis (LCH) can be made based on clinical, radiological, histological (e.g., abundant eosinophilic cytoplasm) and immunocytochemical grounds (e.g., CD1a, CD68, CD163, CD207), although molecular genetics are likely to aid this process [456–458]. An early cytogenetic analysis of LCH reported del(7q22), del(7q22), −15, −16, −17, −18, t(2;4)(p21;q33), t(9;19)(q12; q13), and inv(13)(q21;q33) [459]. A genetic analysis of 61 cases revealed that 57% had *BRAF* ^V600E^ [457], while others noted altered *MAP2K1* in 27.5% of cases [458] and abnormalities in *KRAS, ARAF, NRAS,* and *CSF1R* (located at 5q32 and coding for the receptor for the cytokine macrophage colony-stimulating factor 1, also known as colony-stimulating factor 1), which may be linked to transformation [460]. This sub-section also includes Langerhans cell sarcoma and indeterminate dendritic cell tumours and sarcomas [91], the precise molecular pathology for which is yet to be determined.

#### Histiocytic Neoplasms

In 1988, Benz-Lemoine [461] and colleagues reported a malignant histiocytosis characterised by t(2;5)(p23;q35), which, with the benefit of 20 years of additional research [462], may have been a variant linked to *ALK* (coding for anaplastic lymphoma kinase at 2p23.2-p23.1), now recognised as an important malignancy [463]. In many cases, *ALK* has gene fusion partners that include *CLTC, COL1A2, DCTN1, EML4, TFG, TPM3,* and *TRIM33* [464]. An important second member of this group is Erdheim-Chester disease, for which the leading genetic lesion is *BRAF* ^*V600E*^, present in approximately 50% of cases [464]. This mutation, therefore, points to the possibility of treatment with an inhibitor of the mitogen-activated protein kinase (RAS-RAF-MEK-ERK) pathway (as is the case with Langerhans cell histiocytosis), although there may also be mutations in *ARAF, MAP2K1, NRAS,* and *PI3KCA* [465]. Pai and colleagues have estimated that Erdheim-Chester disease comprises 37% of all histiocytic disorders, marginally exceeding Langerhans cell histiocytosis at 34% [466]. Other conditions in this sub-section include Rosai-Dorfman disease (most commonly linked to *RAS* isoforms [*KRAS, NRAS*], *MAP2K1,* and *ARAF*), juvenile xanthogranuloma (linked to *RAS* isoforms and *MAP2K1),* and histiocytic sarcoma (linked to alterations in *KRAS, BRAF,* and *MAPK1)* [466–468].

### Routine Molecular Pathology of the MPNs

The National Genomic Test Directory for England [52] does not refer directly to MF, ET, or PV, but it does have extended sections on myeloproliferative neoplasms, on myelodysplasia, and on MDS/MPN, i.e., myelodysplasia/myeloproliferative neoplasm, indicating the possibility of aetiological and clinical overlap. Each section describes a multi-target NGS panel to detect small variants in certain genes, many of which are duplicated, as follows:

- Myelodysplasia: *TP53, SF3B1, IDH1, IDH2, NRAS, KRAS, TET2, SRSF2, ASXL1, DNMT3A, RUNX1, U2AF1, EZH2, BCOR, PTPN11, JAK2, SETBP1, PPM1D, DDX41, PHF6, CUX1, and UBA1.*
- Myeloproliferative neoplasms: *KRAS, NRAS, TP53, JAK2, CALR, MPL, ASXL1, CBL, CSF3R, CUX1, DNMT3A, EZH2, IDH1, IDH2, IKZF1, KIT, NFE2, SF3B1, SH2B3, SRSF2, TET2, U2AF1, HRAS, RUNX1, SETBP1, and ZRSR2.*
- MDS/MPN: *KRAS, NRAS, TP53, JAK2, CALR, MPL, ASXL1, CBL, CSF3R, CUX1, DNMT3A, EZH2, IDH1, IDH2, IKZF1, KIT, NFE2, SF3B1, SH2B3, SRSF2, TET2, U2AF1, HRAS, RUNX1, SETBP1, ZRSR2, BCOR, PTPN11, FLT3, NF1, and NPM1.*

Similarly, karyotyping can be used to detect numerous chromosomal abnormalities, some of which are complex, and again, there is marked repetition. Selected translocations of relevance to myeloproliferative neoplasms are presented in Table 22.

- Myelodysplasia: −7/del7q, −5/del5q, i(17q)/t(17p), −13/del13q, del(11q), del12p/t(12p), del(9q), del17/del17p, and idic(X)(q13); −Y; del(20q); +8; +19, (inv(3)/t(3q)/del(3q).
- The entries for myeloproliferative neoplasm and MDS/MPN are almost identical and include del(4q12), −7/7q, −5/5q, i(17q)/t(17p), −13/13q, del(11q), del12p/t(12p), del(9q), --17/17p and idic(x)(q13), t(9;22)(q34;q11) *BCR::ABL1*, del(4)(q12q12) *FIP1L1::PDGFRA*, other *PDGFRA* rearrangements, t(5;12)(q33;p13) *ETV6::PDGFRB*, other *PDGFRB* rearrangements, *FGFR1* rearrangements, t(8;9)(p22;p24) *PCM1::JAK2*, inv(3), and rearranged *ABL1, JAK2, FLT3, RET,* and *NTRK3.*

**TABLE 22:** Selected translocations in myeloproliferative neoplasms.

Translocation	Fused genes	Translocation	Fused genes
t(1;7)(p32;q11)	*TRB::TAL1*	t(7;12)(q36;p13)	*MNX1::ETV6*
t(1;19)(q23;p13)	*TCF3::PBX1*	t(8;21)(q22;q22)	*RUNX1::RUNX1T1*
t(1;22)(p13;q13)	*RBM15::MRTFA*	t(9;11)(p21;q23)	*MLLT3::KMT2A*
t(3;5)(q25;q34)	*NPM1::MLF1*	t(10;11)(p12;q23)	*KMT2A::MLLT10*
t(4;11)(q21;q23)	*KMT2A::AFF1*	t(10;14)(q24;q11)	*TLX1::TRD*
t(5;11)(q35;p15.5)	*NUP98::NSD1*	t(11;14)(p15;q11)	*TRD::LMO1*
t(5;14)(q35;q32.2)	*BCL11B::TLX3*	t(11;19)(q23;p13.3)	*KMT2A::MLLT1*
t(6;9)(p22;q34)	*DEK::NUP214*	t(11;19)(q23;p13.1)	*KMT2A::ELL*
t(6;11)(q27;q23)	*KMT2A::AFDN*	t(12;21)(p13;q22)	*ETV6::RUNX1*
t(7;10)(q34;q24)	*TRB::TLX1*	t(15;17)(q24;q21)	*PML::RARA*
t(7;11)(p15;p15)	*NUP98::HOXA13*	t(17;19)(q22;p13)	*TCF3::HLF*

From: Molecular Pathology: A Primer for Laboratory Scientists, First Edition.

A Blann. © 2025 John Wiley & Sons, Ltd.

The use of FISH is also common between the three sections to determine copy number variation in chromosomes 7/7q, 5/5q, 13/13q, 11q, 12q, 17/17p, i17q, Y, 20, 8, and 19, for rearrangements in *PDGFRA, PDGFRB, FGFR1, FLT3, RET, NTRK3, JAK2, TAL1, TLX1, ABL1, KMT2A, CSF1R, IGH, CRLF2, EPOR, NUP98, BCL2, BCL6,* and *KMT2A,* and for inversions such as inv(3)(q21q26), bringing together *GATA2-MECOM*, inv(16)(p13.1q22) *CBFB-MYH11*, and inv(16)(p13.3q24.3) *CBFA2T3-GLIS2*.

The Directory’s section on histiocytosis describes a multi-target NGS panel for the detection of small variants in *BRAF, MAP2K1, NRAS, KRAS, HRAS, ERBB3, ARAF, MAP3K1, PIK3CA,* and *PIK3CD*. A second panel may be used for detecting structural variants in *EML4-ALK, TPM3-ALK, KIF5B-ALK, MIGA1-BRAF, PACSIN2-BRAF, RNF11-BRAF, CLIP3-BRAF, LMNA-NTRK1, TPR-NTRK1, ETV3-NCOA2*, and rearrangements of *ALK, BRAF, and NTRK1*. Structural variants of many of the latter may also be detected by FISH/PCT. The importance of molecular genetics, as in many cases, lies in informing treatment decisions, such as the use of vemurafenib and binimetinib for *BRAF* ^*V600E*^.

### Non-Coding RNAs in MPNs

Compared to other blood cancers, the literature on non-coding RNAs in MPNs is limited. Nevertheless, lessons learned from other conditions are likely to be informative, such as ncRNA involvement in the biology of JAK/STAT signalling, and in normal and malignant haematopoiesis [469–473].

#### MPN Comparison

In several instances, clinical studies have not specified or have merged the three major MPN groups into a single cohort with varying results [474–476], although others have presented parallel analyses. Hussein and colleagues reported the differential expression of 365 miRNAs in megakaryocytes from 18 cases of pMF, 18 cases of ET, and 8 cases of megakaryocyte hyperplasia, all controlled by 5 cases of normal haematopoiesis. The leading result was increased expression of miR-146b in pMF alone [477], although such small sample sizes raised concerns about false positives and negatives. Tombak et al. investigated six peripheral blood miRNAs in 22 cases of pMF, 22 cases of PV, 49 cases of ET and 40 controls. The principal findings were increased expression of miR-155 and reduced expression of miR-451 in all three disease groups, increased expression of miR-221 in pMF and ET, lower expression of miR-222 in PV, but higher expression of miR-222 in ET, and increased expression of miR-223 in pMF and ET. Although ET and pMF patients had higher levels of miR-223, there was no difference in the expression of miR-181a [478]. Afar and colleagues studied bone marrow from 40 cases of PV, 27 cases of ET, 40 cases of MF secondary to PV or ET, and used peripheral blood from 90 subjects as a control group. Their primary result was an increased frequency of the TT genotype in miR-146a rs2431697 in MF, with an increased frequency of the expression of *IL-1β* in the TT genotype in all three groups compared to the controls. However, the TT genotype was not linked to the transformation of PV or ET to MF [479]. Several research groups have reported miRNAs in each of the three major disease groups.

#### Polycythaemia Vera

Bruchova and colleagues studied changes in the expression of miR-150, miR-155, miR-221, miR-222, miR-451, miR-16, miR-339, and miR-378 in peripheral blood mononuclear cells expanded *in vitro* by growth factors [480]. They found a positive correlation between the frequency of *JAK2 V617F* and the expression of miR-143, together with an inverse correlation with let-7a, miR-30c, miR-342 and miR-150 [481]. Data from Guglielmelli et al. suggest that deregulation of miR-16–2 contributes to the abnormal expansion of the erythroid lineage in PV [482]. Zhan and colleagues [483] probed circulating CD34-positive cells in 8 PV patients and 6 healthy controls, finding that 71 miRNAs were either up- or downregulated, hypothesising that four core species may act on target genes and thus have an effect on malignant transformation:

- miR-575, targeting *SFRS2, SFRS1, EPOR, HMGA2,* and *TFPI*,
- miR-887, targeting *GSK3A* and *BIM*,
- miR-196b, targeting *HOXA5, HOXA7, HOXA9, HOXA10, HOXB6, HOXB7, HOXC8, HMGA2,* and *ERG*,
- miR-551b, targeting *ERBB4.*

#### Essential Thrombocythaemia

Navarro and colleagues extracted total RNA from the platelets of 19 patients with ET and 10 controls, quantifying the expression of 384 mature miRNAs. Of these, the ET samples showed a distinct signature of 70 species, 68 of which were downregulated compared to the control samples (with miR-9 and miR-431 being upregulated). Forty miRNAs differed between ET patients depending on their *JAK2* status (mutated or wild type), 8 of which were proposed as being likely to activate the JAK/STAT pathway via *SOCS1* and *SOCS3* [484]. Trans et al. also probed platelets, finding that miR-10a, miR-28, miR-126, miR-155, miR-221, miR-222, miR-223, and miR-431 were downregulated, and that miR-9 (confirming data from Navarro et al) and miR-490 were upregulated compared with healthy controls. The expression of certain miRNAs correlated with metrics of platelet biology, such as mean platelet volume and P-selectin expression [485].

#### Myelofibrosis

Calura et al. used NGS to profile 584 miRNAs and 18,654 genes in 73 CD34^+^ cells from 42 cases of pMF, and from 31 controls, ordering them into a large series of interacting networks with potential targets. Among the many observations were links between key genes that code for transcription factors (*MYCN, ATF, CEBPA, REL, IRF,* and *FOXJ2*) and miR-106a-5p, miR-20b-5p, miR-20a-5p, miR-17-5p, miR-19b-3p, and let-7d-5p [486]. Not only did Norfo et al. find 58 differentially expressed genes in pMF (with *DEFA1* showing a 60-fold increase), they also observed changes in numerous miRNAs, describing gene/miRNA networks. The strongest interaction in these networks was found to be between miR-155-5p and tumour suppressor *JARID2* at 6p22.3, with implications for megakaryocyte hyperplasia [487]. Rontauroli and colleagues suggested a role for upregulated miR-494-3p in megakaryocyte hyperplasia via an interaction with *SOCS6* at 18q22.2, coding for suppressor of cytokine signalling 6 [488]. Similarly, Fuentes-Mattei et al. showed that miR-543 is increased in pMF and that it targets *TET1* and *TET2*, both coding for enzymes with roles in the epigenetic regulation of DNA [489].

#### Myelodysplasia

As discussed above, mutations in *ASXL1* are a common feature of MDS (and other blood cancers). One such mutation causes an upregulation of miR-125a, which in turn leads to the repression of *Clec5a* at 7q34, coding for a lectin-like molecule with links to *SYK*, a tyrosine kinase [490]. Micheva and Atanasova summarised the potential diagnostic and prognostic roles of nine miRNAs in MDS, with the upregulation of miR-22 possibly involved in transformation by interacting with *TET2* and *PTEN*. The authors also pointed to 9 upregulated and 13 downregulated species linked to del(5q), along with 15 with implications for therapy [491]. Many of these miRNAs were also described by Georgoulis and colleagues, who highlighted those of prognostic value together with other ncRNAs (circular, long non-coding RNAs, small nucleolar RNAs, and Piwi-interacting RNAs) [492].

### Summary: The Complex Nature of MPNs

The section highlighted the complexity of the clinical aspects, cell biology, and molecular genetics of this group of diseases, with the potential for any number of genes to have a place in leading to the development of these conditions [91]. Despite this, a small number of general conclusions can be drawn, as indicated in Table 23. Overall, the leading mutation (in terms of number of cases with these specific diseases) is *JAK* ^*V617F*^, prevalent in PV, ET and pMF, the aberrant signalling of which explains many of the associated transformations, and in many cases, there is a place for cytokine growth factors [493–495]. While the histocytoses also have several subtypes, the primary focus is on *BRAF* ^*V600E*^ and *ALK* [459, 468, 496]. The role of the tumour suppressor *VHL* in Chuvash polycythaemia and erythrocytosis [353, 497] provides an alternative pathophysiological mechanism to that observed in other cancers, such as in certain renal cell carcinomas [498], in which the gene is inactivated in 85% of cases. Future research may clarify many of these issues (such as the sensitivity and specificity of a particular lesion), while the relatively recent development of CHiP may also provide further insight [499–501].

**TABLE 23:** Frequency of leading gene mutations in MF, ET and PV.

Gene	PV	ET	pMF
*JAK2*	98%	55%	60%
*MPL*	0%	5%–7%	7%–10%
*CALR*	0%	25%–30%	20%–30%
*DNMT3A*	5%–10%	1%–5%	8%–12%
*IDH1*	1%–2%	1%–2%	5%–6%
*IDH2*	1%–2%	1%–2%	5%–6%
*ASXL1*	2%–7%	5%–10%	15%–35%
*EZH2*	1%–2%	1%–2%	7%–10%
*NRAS*	<2%	<2%	2%–4%
*KRAS*	<2%	<2%	2%
*CBL*	<2%	<2%	4%
*SRSF2*	<2%	<2%	6%–14%
*U2AF1*	<2%	<2%	7%–10%
*TP53*	<2%	<2%	2%–5%
*TET2*	10%–20%	1%–3%	2%–4%
*SH2B3*	2%–9%	1%–3%	2%–4%
*RUNX1*	<2%	<2%	2%–3%

PV, polycythaemia vera; ET, essential thrombocythemia; pMF, primary myelofibrosis. From: Molecular Pathology: A Primer for Laboratory Scientists, First Edition. A Blann. © 2025 John Wiley & Sons, Ltd.

## Paediatric Cancers

### Introduction and Epidemiology

There is considerable evidence that the aetiology of many paediatric cancers, especially those of a haematological nature, differs from that of adult cancers [500–502]. While high mutational burdens are often present in adult malignancies, possibly arising from a long latency period and/or an increased rate of lesion development, those in young patients are more likely to be driven by a fusion oncogene, a single gene driver, or alterations in gene structure or copy number, which may have occurred *in utero* [503–506].

From 2001 to 2010, globally, blood cancers were the most frequent malignancy in children aged 0 to 4 (∼42%), 5 to 9 (∼49%), 10 to 14 (∼43%) 15 to 19 (∼38%) year-olds, with age-standardised rates of 46.4 per million person-years for leukaemia and 15.2 for lymphoma [for comparison, 28.2 for central nervous system (CNS) tumours] [507]. Data from the Office of National Statistics reported 16 deaths from AML and 25 from ALL in England and Wales in 2023 in those aged up to 14, with a significant difference in age distribution (Table 24) [1]. Notably, the 12 deaths due to ALL at ages 10–14 were surpassed only by those deaths at age 55–59 years, whereas deaths due to AML were exceeded at age 20–24 and point to major differences in the cell biology of these diseases. The molecular pathology of AML and ALL, focusing on adult disease, has already been described.

**TABLE 24:** Deaths from leukaemia in England and Wales in 2023.

​	Age group (years)			
	<1	1–4	5–9	10–14
AML	1	9	3	3
ALL	0	5	8	12

### ALL

In children, ALL comprises 75%–80% of malignancies, and in turn, 85% of which are B cells. Leading chromosome abnormalities include hyperdiploidy (often >51 chromosomes in approximately 25% of cases, often with +4. +10, +17, and +21), the primary genetic lesions being (t(9;22)(q34:q11)/*BCR::ABL1*) and t(12;21)(p13;q22)/*ETV6::RUNX1*, detectable by FISH [508]. Other abnormalities include several *PAX* fusions, such as dicentric (dic)(9;12)(p13;p3), which forms *PAX::ETV6*, t(7;9)(q11;13), which forms *PAX::ELN*, t(3;9)(p13;p13), which forms*PAX5::FOXP1*, and t(9;15)(p21;q25), which forms *PAX5::PML* [509]. Mutations in *NRAS, FLT3, KRAS, BAK1, PTPN11, SOS1, NF1, CREBPE, CREBBP, CDKN2A/B, IKZF2/3, PAG1, TP53,* and *RB1* in B-cell ALL have been described, while Haas and Borkhardt consider *ARID5B, CEPBE, BMI1,* and *PIP4K2A* to be the four most relevant susceptibility loci [510, 511]. The most frequently mutated genes in T-ALL include *TLX3, TAL1, LMO1, HOXA* locus, *KMT2A, LMO2,* and *MYC* [512]. Approximately 3%–5% of paediatric ALL cases carry the t(9;22)(q34:q11,2)/*BCR::ABL1* Philadelphia chromosome [513].

### AML

AML is also a genetically heterogeneous disease. The main chromosomal abnormalities include t(8;21)(q22;q22)/*RUNX1::RUNX1T1* (the most common, present in ∼15%), and inv(16)/t(16;16)(p13;q22)/*CBFβ::MYH11* (10%–15%) [514]. Gene rearrangements include those in *MLL* at 11q23, with SNPs or copy number variation in *KMT2A* (present in 16%–20% of cases)*, FLT3* (10%–20%), *NPM1* (∼10%), *CEBPA* (5%–10%), *WT1* (15%), *KIT, KRAS, NRAS, NUP98, PTPN11, RUNX1,* and *TP53* (all ≤5%), with fusions (such as *CBFA2T3::GLIS2)* being more common in paediatric AML (80% of cases) than in adult AML (53%) [514–517]. Although several of these genes are also linked to ALL, there are major differences – the frequency of a normal cytogenetic profile in AML is 21%, compared to only 6% in ALL; the frequencies of one, two or three or more alterations are 50%, 16% and 13% in AML, and 26%, 22% and 46% in ALL, respectively [515].

### Acute Promyelocytic Leukaemia

This is characterised in many cases (>90%) by t(15;17)(q24;q21)/*PML::RARA*, this condition is present in 5%–10% of paediatric AMLs, while 8-year survival rates are close to 100%, leading to treatments (as in adult disease) being all-trans-retinoic acid and arsenic trioxide [514, 517, 518]. Other rare abnormalities include *TBL1XR1::RARB*, *KMT2A::SEPT6* and *NPM1::RARA* fusions [519]. In England and Wales from 2013 to 2023, there was a single death in the 0–14 age group due to this disease, and seven in the 15–19 age group [1].

### Juvenile Myelomonocytic Leukaemia

The leading drivers of this rare (1%–2% of paediatric leukaemias) MDS/MPN overlap include *PTPN11* (32%–41%)*, NRAS* (15%–17%)*, KRAS* (17%–18%)*, NF1* (5%–13%)*,* and *CBL* (11%–17%), one or more of which are present in 90% of cases. Mutations may also be found in genes such as *ALK* and *ROS1,* while fusions leading to *FIP1L1::RARA, HCMOGT1::PDGFRB, NDEL1::PDGFRB,* and *NUP98::HOXA11* may also be present [520, 521]. In England and Wales from 2013 to 2023, there were five deaths in the 0–19 age group due to this disease, all in the 1 – 4 age group (4 deaths) and the 5 – 9 age group (1 death) [1].

The National Genomic Test Directory for England describes a multi-target NGS panel for detecting small variants in *KRAS, NRAS, TP53, JAK2, CALR, MPL, ASXL1, CBL, CSF3R, CUX1, DNMT3A, EZH2, IDH1, IDH2, IKZF1, KIT, NFE2, SF3B1, SH2B3, SRSF2, TET2, U2AF1, HRAS, RUNX1, SETBP1, ZRSR2, BCOR, PTPN11, FLT3, NF1,* and *NPM1*, in addition to investigating chromosomes 5, 7, and 8 using FISH [52].

### Lymphomas

Principally NHLs, Derebras and colleagues characterised paediatric lymphomas, which broadly reflect the full range of NHLs in adults [522], with an annual incidence of ∼80/million in children aged ≤19, with the most common being BL [22], DLBCL [18], and lymphoblastic lymphoma (12/million). Poor prognosis in DLBCL is linked to *MYC* 8q25 rearrangements, t(14;18)(q32;q21)/*IGH::BCL2*, and *BCL6* 3q27 rearrangements, while good prognosis is linked with t(6;14)(p25;q32)/*IGH::IRF4*. Similarly, in Burkitt lymphoma, poor prognosis is linked to *c-MYC* translocations and immunoglobulin genes *IgH, IgK* and *IgL*, i.e. t(8;14)(q24;q32), t(8;22)(q24.1;q11.2), and t(2;8)(p12;q24.1), respectively, in addition to del(13q14.3) or del(13q34). In Burkitt-like lymphoma, an 11q aberration with proximal gains and telomeric losses indicates a favourable outcome, while the leading abnormality in anaplastic large cell lymphoma is t(2;5)(p23;q35), forming *NPM1::ALK*.

The 2022 WHO classification of haematolymphoid tumours mentions paediatric marginal zone lymphoma and paediatric-type follicular lymphoma (ptFL), but there is no reference to associated genetic or chromosomal abnormalities, and no mention of any other paediatric blood cancers [25]. However, ptFL differs from “adult” FL in that there is a high frequency of *MAP2K1* mutations and 1p36 alterations, the location of *TNFRS14* (CD270, a member of the TNF superfamily that can mediate herpes simplex virus entry into the cell), alongside mutations in tumour suppressor *IRF8* [523]*.*

### Histiocytosis

The leading condition in this group is Langerhans cell histiocytosis, presenting in 5–9 cases per million, compared to 0.07 per million in individuals over the age of 18. This is most frequently associated with *BRAF* ^*V600E*^, and if present, guides treatment directed towards the RAS-RAF-MEK-ERK-MAP kinase pathway [459, 496, 524–526]. The rarity of juvenile xanthogranuloma brings numerous problems regarding presentation, diagnosis, and management, although the absence of *BRAF* ^*V600E*^ is important, whereas alterations in *MAPK21, KRAS*, and *NRAS* may be present in 20%–30% of cases: other candidate genes include *NF1, NF2, ARAF,* and *PI3KCD* [468, 527].

### Summary of Paediatric Blood Cancer

Several indicators show that many of the paediatric blood cancers, especially leukaemias, differ fundamentally from adult cancer. These are led by ALL, which is often characterised by hyperdiploidy, the Philadelphia chromosome, and t(12;21)(p13;q22)/*ETV6::RUNX1*, although one (or more) of many individual gene lesions may be present. In AML, the leading chromosomal lesions are t(8;21)(q22;q22) and inv(16)/t(16;16)(p13;q22), while mutated *KMT2A, NPM1, CEBPA,* and *WT1* are among the most common genetic abnormalities. As with adult acute promyelocytic leukaemia, almost all paediatric cases are characterised by t(15;17)(q24;q21)/*PML::RARA*, and are highly treatable. Approximately 90% of cases of the very rare juvenile myelomonocytic leukaemia are linked to abnormalities in the *PTPN11, NRAS, KRAS, NF1,* and *CBL genes*. Paediatric lymphoma can generally be grouped in the same way as adult disease, with an aetiology focusing on *MYC* and immunoglobulin genes, although del(13) and −11q variants are well known, as are abnormalities in *BCL6, IRF4* and *TP53.* Paediatric follicular lymphoma is often linked to *MAP2K1* mutations, while the principal histiocytosis of Langerhans cells is most frequently associated with *BRAF* ^*V600E*^.

## Conclusion

Molecular pathology plays a key role in blood cancers and in non-malignant haematological diseases [528]. As with all malignant neoplasms, those affecting the blood are associated with an ever-expanding genetic signature ranging from causative to coincidental. As NGS and other molecular approaches become more common, the incorporation of these genetic signatures into the diagnosis, prognosis and treatment of patients will also become more prevalent in the coming years. While great progress has been made, there is still significant work to do to elucidate the complex relationships between molecular signatures and certain neoplasms and to incorporate them into clinical practice. However, the potential advantages are profound, as they will allow precise diagnoses and personalised treatment plans to be developed.
